# Supplementary material for: Light-gated redox switching and actuation in polymer hydrogels
Source: Nat Commun. 2025 Oct 14;16:9106. doi: 10.1038/s41467-025-64123-5 (PMC12521516; doi:10.1038/s41467-025-64123-5)
Supplement: Supplementary file 1 — Supplementary Information [file 41467_2025_64123_MOESM1_ESM.pdf]

Supplementary Information

Light-gated Redox Switching and Actuation in Polymer Hydrogels

Roza R. Weber[1], Robert Hein[1,2,*], Alexander Ryabchun[1], Yohan Gisbert[1,3], David Garcia Romero[4], Maria Antonietta Loi[4], and Ben L. Feringa[1,4,*]

1) Stratingh Institute for Chemistry, University of Groningen, Nijenborgh 4, 9747AG Groningen, The Netherlands
2) Organic Chemistry Institute, University of Münster, Corrensstraße 40, 48149 Münster, Germany
3) Current address: Univ Rennes, CNRS, ISCR − UMR 6226, F-35000 Rennes, France
4) Zernike Institute for Advanced Materials University of Groningen Nijenborgh 4, 9747 AG Groningen, The Netherlands

*robert.hein@uni-muenster.de, *b.l.feringa@rug.nl

Contents

Supplementary Methods and Discussions 2
1 General Remarks 2
2 Synthesis and Characterization 3
3 UV/Vis studies 10
3.1 Photoswitching 10
3.2 Oxidation & Reduction 11
4 Electrochemistry, Redox Mechanisms and Theoretical Calculations 11
4.1 General Electrochemical Characterization 11
4.2 Mechanism of Redox Switching 14
4.3 Mechanism for Change in Redox Potential Upon Irradiation 21
5 Material Optimization & Rheology 26
6 Swelling 27
7 Actuation 32
8 Fluorescence 35
9 Profilometry 36
10 Patterning 37
11 Supplementary References 38

Supplementary Methods and Discussions

1 General Remarks

All reactions were performed under nitrogen atmosphere unless otherwise noted. Flash column chromatography was performed on silica gel Davisil LC60A (Merck type 9385, 230400 mesh), a Reveleris X2 Flash Chromatography system from Büchi or a Biotage Selekt system (MPLC) using the indicated solvents. NMR spectra (^{1}H and ^{13}C) were recorded on a Varian Mercury-Plus 400 (400 MHz), or a Bruker Avance Neo 600 (600 MHz) spectrometer at 298K (referenced to residual solvent signal: for ^{1}H detection, δ =7.26 ppm ; for ^{13}C detection, δ = 77.16 ppm for $CDCl_3$). High resolution mass spectroscopy (HRMS) was performed on a LTQ Orbitrap XL spectrometer with electrospray ionization (ESI) as ionization technique. Rheological tests were performed on a TA instruments Discovery HR-2 rheometer. LEDs and power meter were purchased from Thorlabs (M455L4, M365L2, PM400 with sensor S401C).

2 Synthesis and Characterization

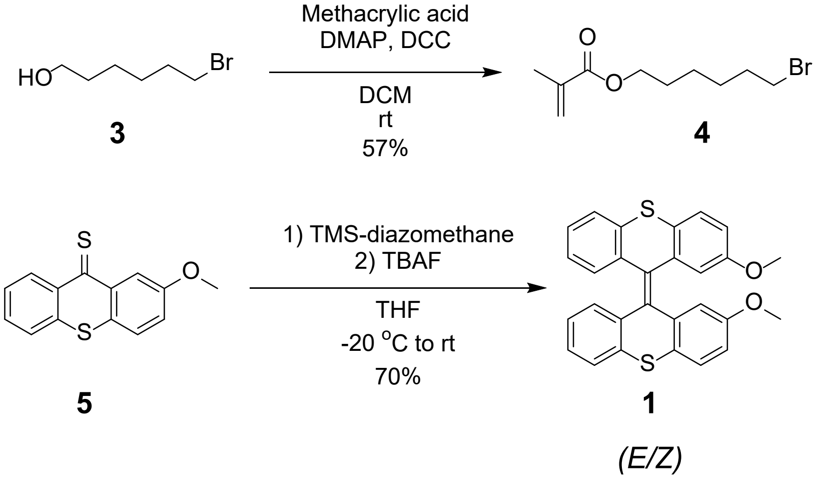

Supplementary Figure 1: Synthetic scheme of the synthesis of compound **1**.

1

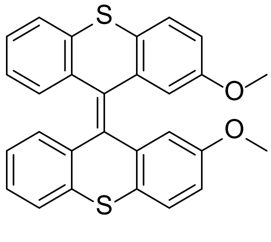

(E/Z)

Synthesised according to a modified procedure[1]: 2-methoxy-9H-thioxanthene-9-thione (2.4 g, 9.9 mmol) was dissolved in 70 mL dry THF (0.04 M). Of this solution, 60 mL was removed using a syringe and stored under nitrogen. The remaining 60 mL was cooled to -20 °C using an NaCl/ice bath. $TMSCHN_2$ (2.7 mL, 5.4 mmol, 0.55 equiv., 2.0 M in hexanes) was added and the mixture was allowed to warm to room temperature, resulting in a colour change from green/black to a clear, light yellow solution. The 60 mL solution of 2-methoxy-9H-thioxanthene-9-thione that was removed earlier was added back to the reaction flask and the resulting mixture was stirred at room temperature for 30 min. 1.0 M in THF tetrabutylammonium fluoride solution (20 mL, 20 mmol, 2.0 equiv.) was added, and the solution was left to stir for ten min. After concentration under reduced pressure the crude product was recrystallized twice from EtOAc. Spectroscopic data is in accordance with literature.[1]

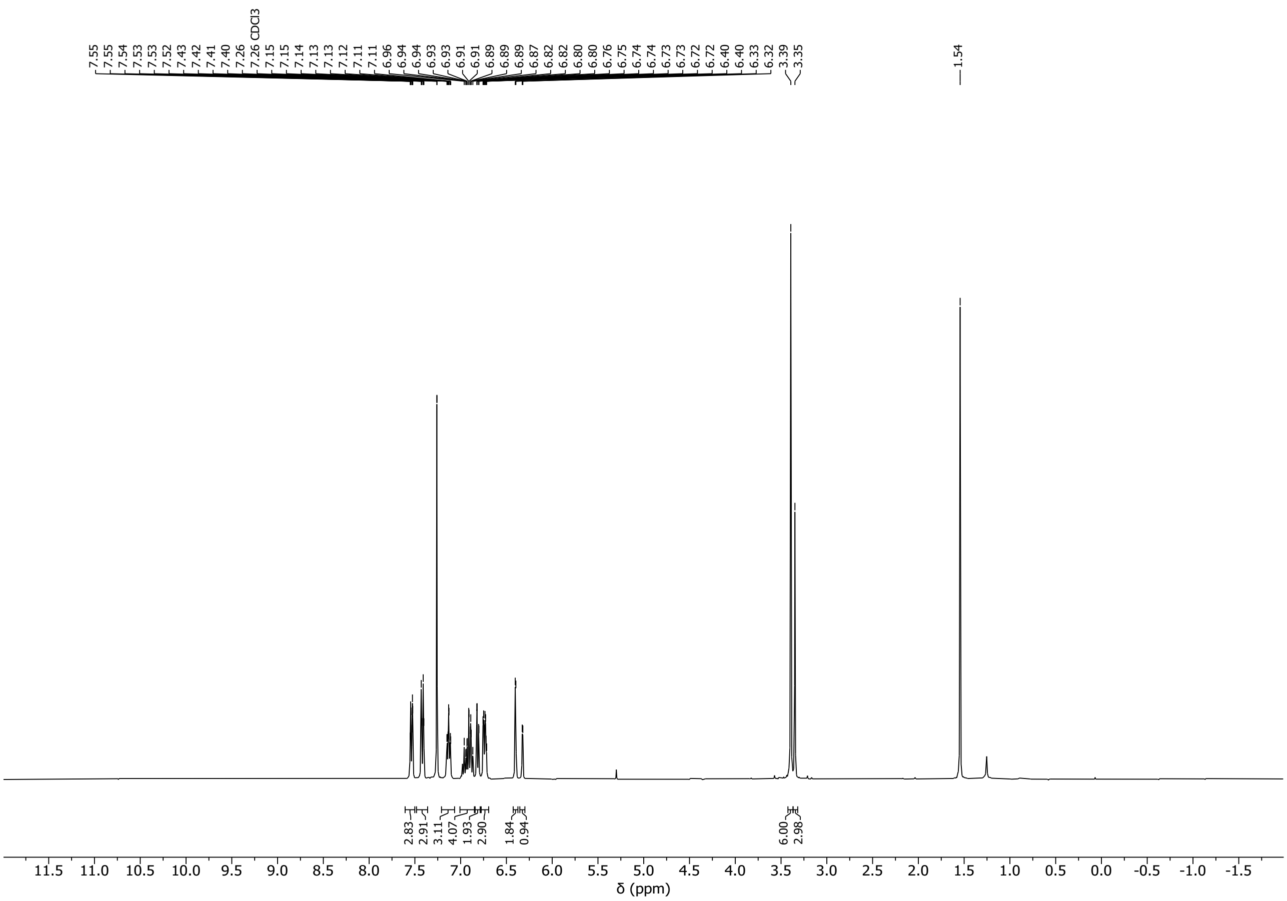

Supplementary Figure 2: ^{1}H NMR spectrum of compound **1**. 400 MHz in $CDCl_3$.

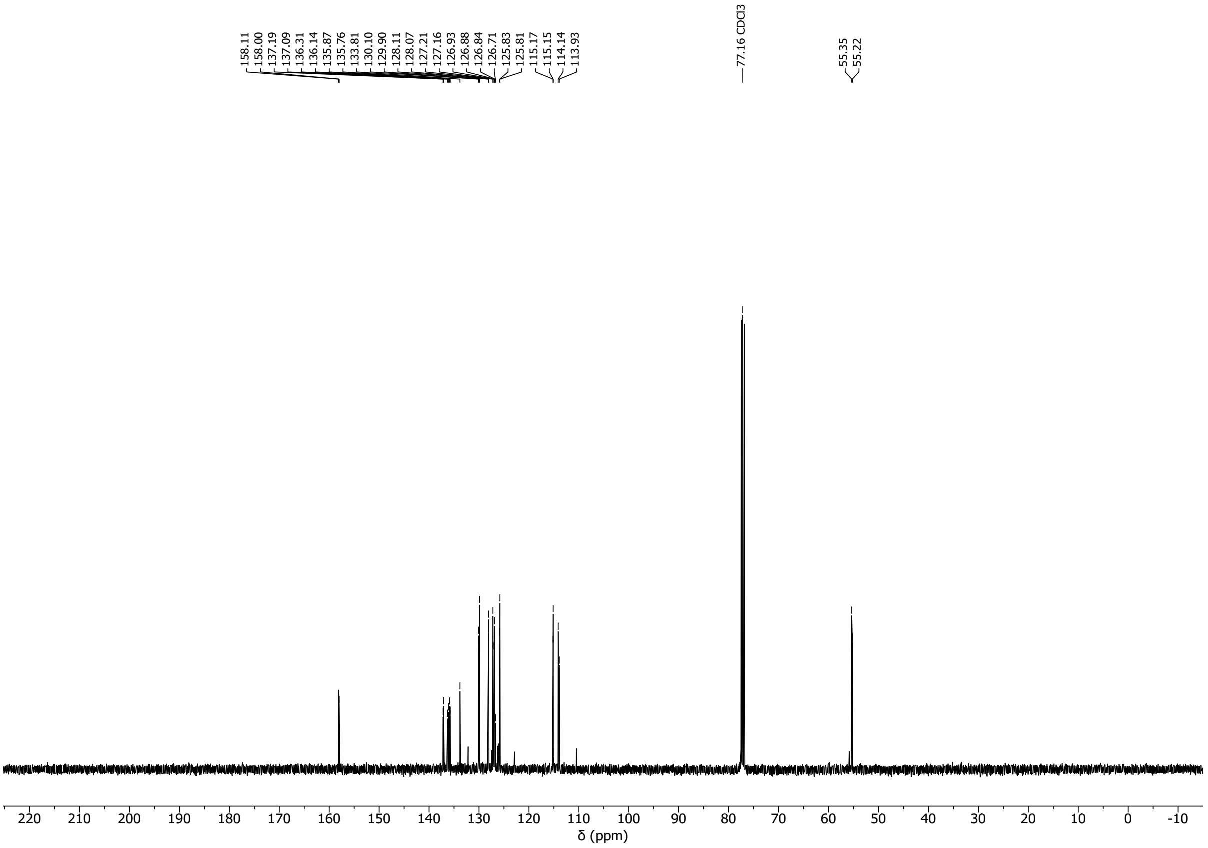

Supplementary Figure 3: ^{13}C NMR spectrum of compound **1**. 101 MHz in $CDCl_3$.

2

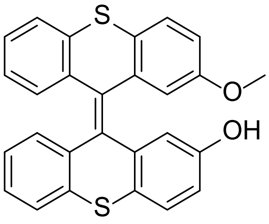

(E/Z)

To a stirred solution of **1** (2.5 g, 5.4 mmol) in 54 mL methylene chloride, BBr_3 solution (1 M in methylene chloride, 3.6 mL, 3.6 mmol, 0.7 equiv.) was added at 0 °C. The solution was left to stir at room temperature for 24 hr, after which it was quenched with water. The aqueous layer was extracted with methylene chloride (3 x 50 mL) and ethyl acetate (3 x 50 mL) to ensure that all BTX species are extracted. The combined organic layer was washed with brine, dried with $MgSO_4$ and concentrated in vacuo. The product mixture was dry-loaded on celite and purified by column chromatography (silica gel, pentane:CH_2Cl_2:MeOH 100:0:0 to 50:50:0 to recover **1** (29%) 50:50:0 to 0:100:0 to isolate both isomers of **2** (together 30%, 56:44 major:minor) and 0:100:0 to 0:90:10 to isolate the diphenolic species (fully demethylated) (29%).

^{1}H NMR (400 MHz, $CDCl_3$) δ 7.58 – 7.49 (m, $2H_{major}$ + $2H_{minor}$), 7.45 – 7.37 (m, $2H_{major}$ + $2H_{minor}$), 7.18 – 7.09 (m, $2H_{major}$ + $2H_{minor}$), 7.01 – 6.83 (m, $3H_{major}$ + $3H_{minor}$), 6.83 – 6.63 (m, $3H_{major}$ + $3H_{minor}$), 6.43 (d, J = 2.7 Hz, $1H_{minor}$), 6.35 (d, J = 2.7 Hz, $1H_{minor}$), 6.30 (d, J = 2.7 Hz, $1H_{major}$), 6.27 (d, J = 2.7 Hz, $1H_{major}$), 4.67 (s, $1H_{major}$ + $1H_{minor}$), 3.42 (s, $3H_{minor}$), 3.34 (s, $3H_{major}$). Product was too insoluble for ^{13}C NMR.

HRMS (ESI-): Calculated: $C_{27}H_{17}O_2S_2^-$ [M-H]$^-$ 437.0675, found : 437.0670.

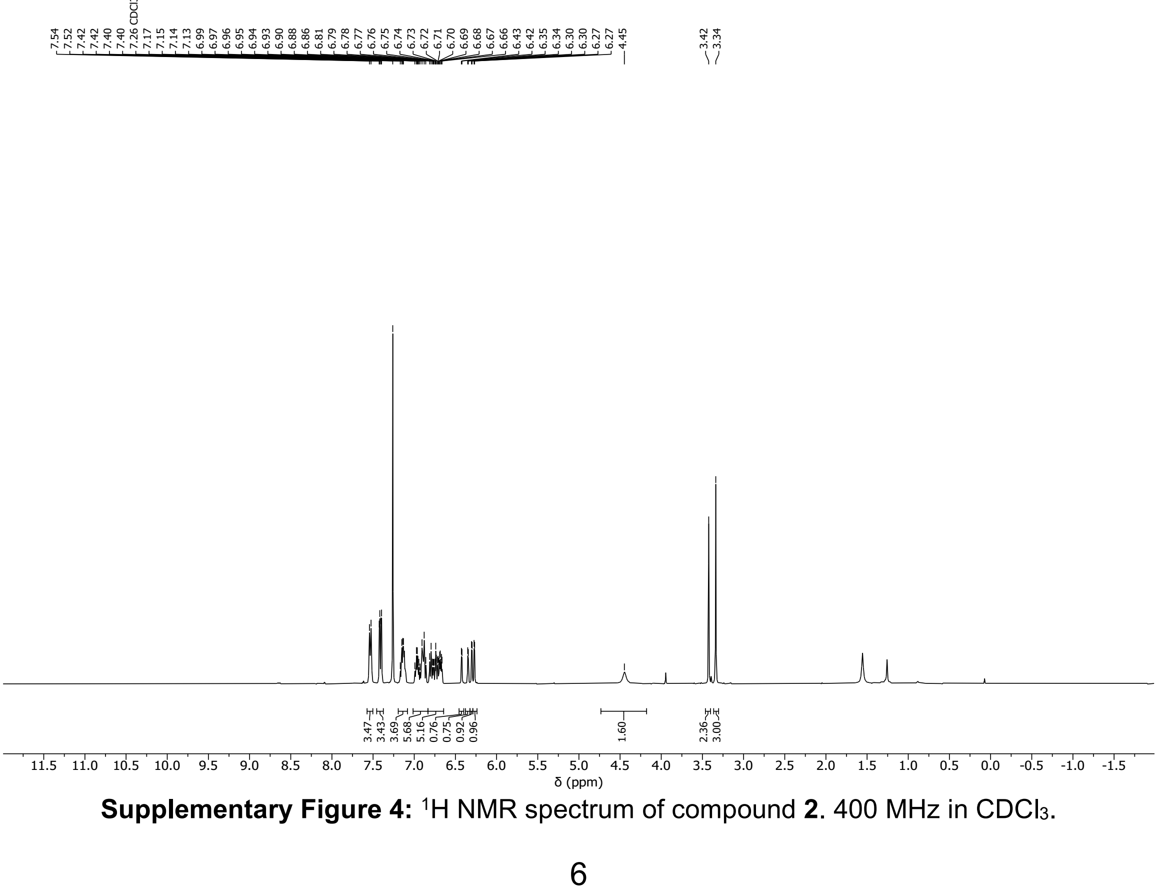

Supplementary Figure 4: ^{1}H NMR spectrum of compound **2**. 400 MHz in $CDCl_3$.

BTX-MA

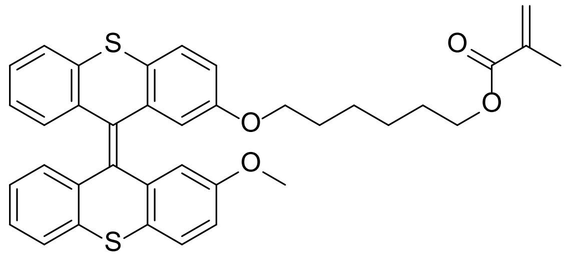

(E/Z)

Compound **2** (50 mg, 0.11 mmol), Cs_2CO_3 (41 mg, 0.13 mmol, 1.1 equiv.) and 6-bromohexyl methacrylate (28 mg, 0.11 mmol, 1 equiv.) were dissolved in 1 mL acetone and heated to 60 °C for 16 hr while stirring. After cooling to room temperature, water was added and the mixture was extracted with CH_2Cl_2 (3 x 10 mL). The combined organic layer was then washed with brine, dried with $MgSO_4$ and loaded on celite. Compound **3** was purified by column chromatography (silica gel, pentane:CH_2Cl_2 90:10:0 to 50:50:0) to give 45 mg of product (mixture of isomers, 60/40 major/minor) as an off-white solid (Yield 65%). When storing this compound for extended periods of time, we recommend adding a small amount of BHT to inhibit polymerization.
^{1}H NMR (400 MHz, $CDCl_3$) δ 7.54 (m, 2H_{major} + 2H_{minor}), 7.46 – 7.35 (m, 4H_{major} + 4H_{minor}) (m, 2H_{major} + 2H_{minor}), 7.17 – 7.08 (m, 2H_{major} + 2H_{minor}), 7.00– 6.85 (m, 3H_{major} + 3H_{minor}), (m, 1H) (m, 1H_{major} + 1H_{minor}), 6.77 – 6.68 (m, 2H_{major} + 2H_{minor}), 6.45 – 6.36 (m, 1H_{major} + 1H_{minor}), 6.36 – 6.25 (m, 1H_{major} + 1H_{minor}), 6.10 (overlapping s, 1H_{major} + 1H_{minor}), 5.55 (overlapping s, 1H_{major} + 1H_{minor}), 4.21 – 4.10 (m, 2H_{major} + 2H_{minor}), 3.68 – 3.53 (m, 1H_{major} + 1H_{minor}), 3.38 (s, 2H_{major} + 2H_{minor}), 3.34 (s, 2H_{major} + 2H_{minor}), 3.33 – 3.25 (m, 1H_{major} + 1H_{minor}), 1.95 (overlapping s, , 3H_{major} + 3H_{minor}), 1.74 – 1.63 (m, 2H_{major} + 2H_{minor}), 1.62 – 1.51 (m, 2H_{major} + 2H_{minor}), 1.41 – 1.33 (m, 4H_{major} + 4H_{minor}).
^{13}C NMR (151 MHz, $CDCl_3$) δ (major and minor signals strongly overlapping) 167.7, 158.1, 158.0, 157.6, 157.6, 137.2, 137.2, 137.1, 137.0, 136.6, 136.3, 136.2, 136.1, 135.9, 135.9, 135.9, 135.8, 133.9, 133.7, 130.1, 130.1, 129.9, 129.9, 128.1, 128.1, 128.1, 128.0, 127.2, 127.2, 127.2, 126.9, 126.9, 126.8, 126.7, 126.7, 126.5, 125.8, 125.8, 125.4, 115.7, 115.6, 115.2, 114.9, 114.7, 114.1, 114.0, 77.4, 77.2, 76.9, 68.0, 67.8, 64.8, 64.8, 55.3, 55.2, 29.0, 28.7, 28.7, 25.8, 25.8, 25.8, 18.5.

HRMS (ESI+): Calculated: $C_{37}H_{34}O_4S_2^+$ $[M]^+$ 606.1893, found : 606.1896.

Elemental analysis (% calcd, % found for $C_{37}H_{34}O_4S_2$): C (73.24, 72.96), H (5.65, 5.98), N (0.00, 0.00)

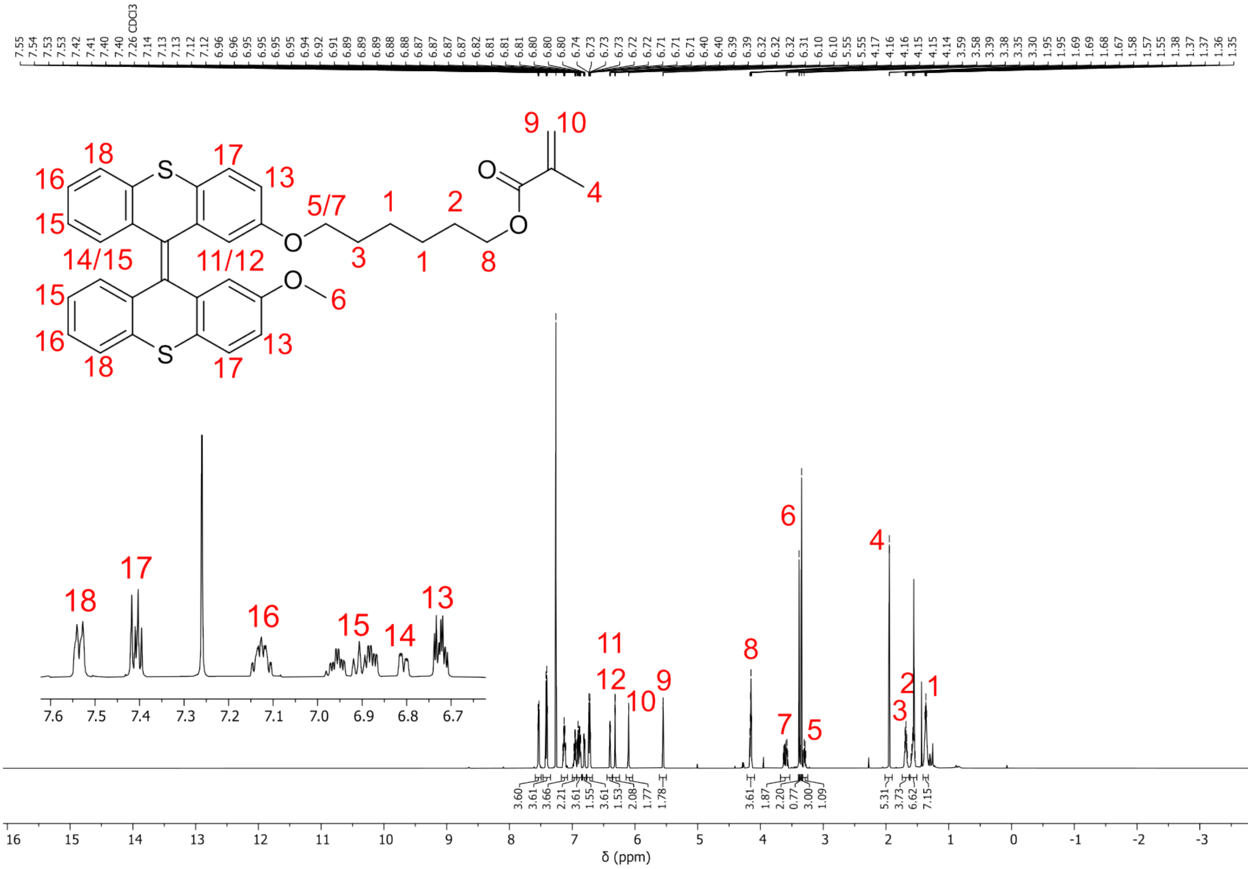

Supplementary Figure 5: ^{1}H NMR spectrum of compound **BTX-MA**. 600 MHz in $CDCl_3$.

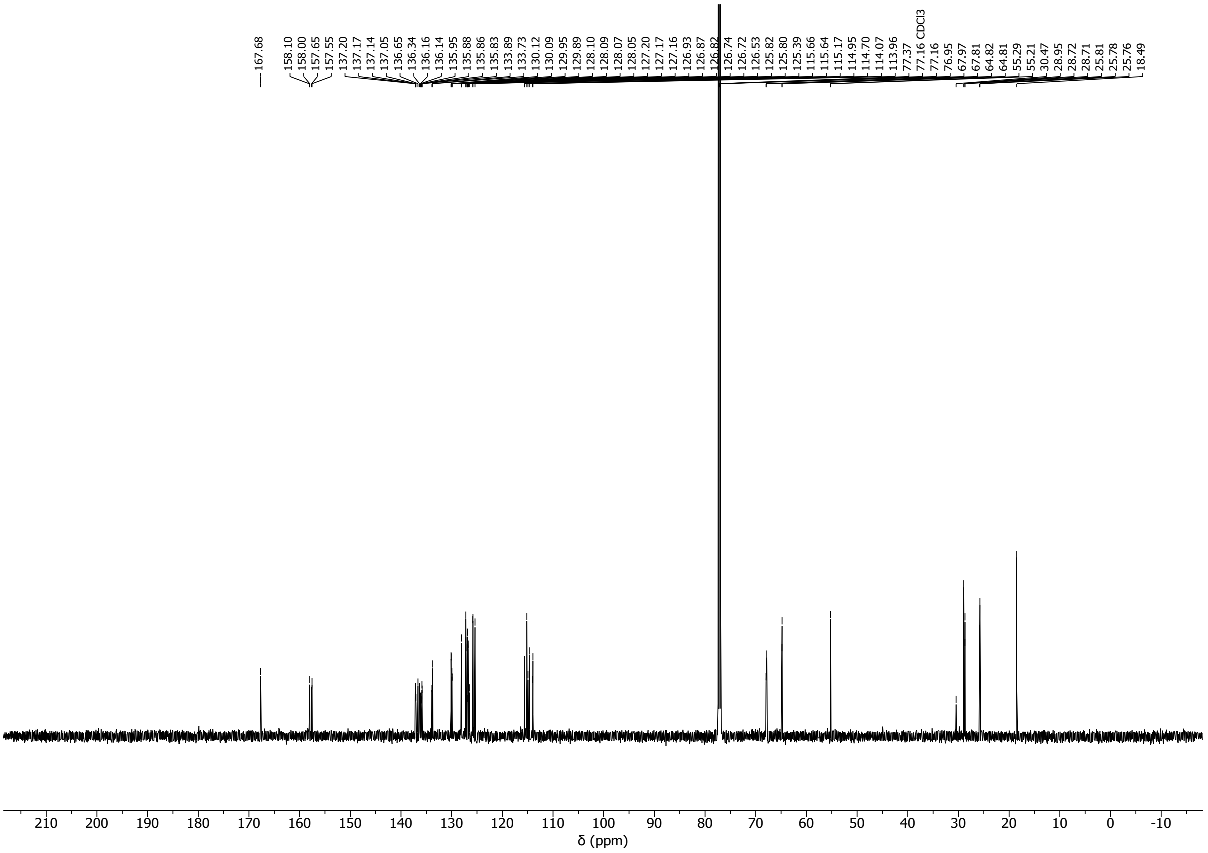

Supplementary Figure 6: ^{13}C NMR spectrum of compound **BTX-MA**. 151 MHz in $CDCl_3$.

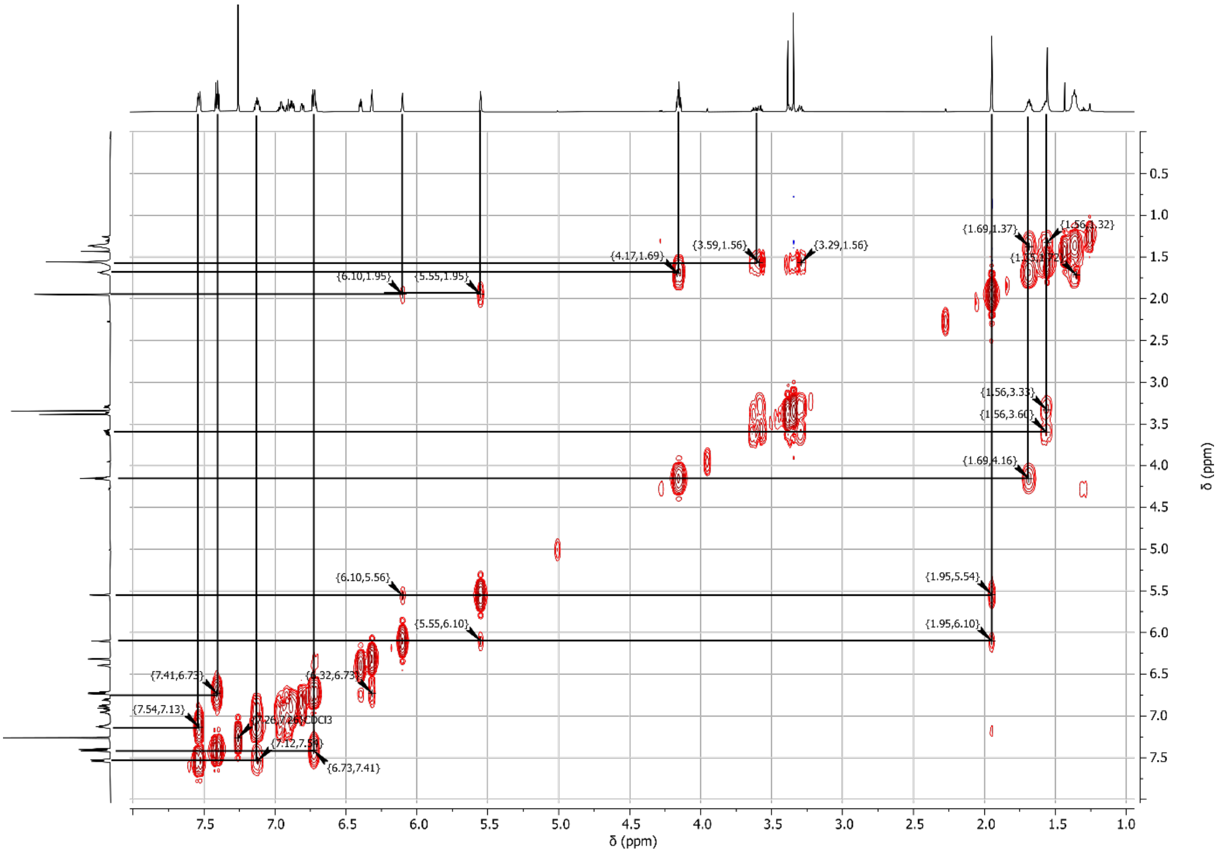

Supplementary Figure 7: COSY NMR spectrum of compound **BTX-MA**. 600 MHz in $CDCl_3$.

3 UV/Vis studies

3.1 Photoswitching

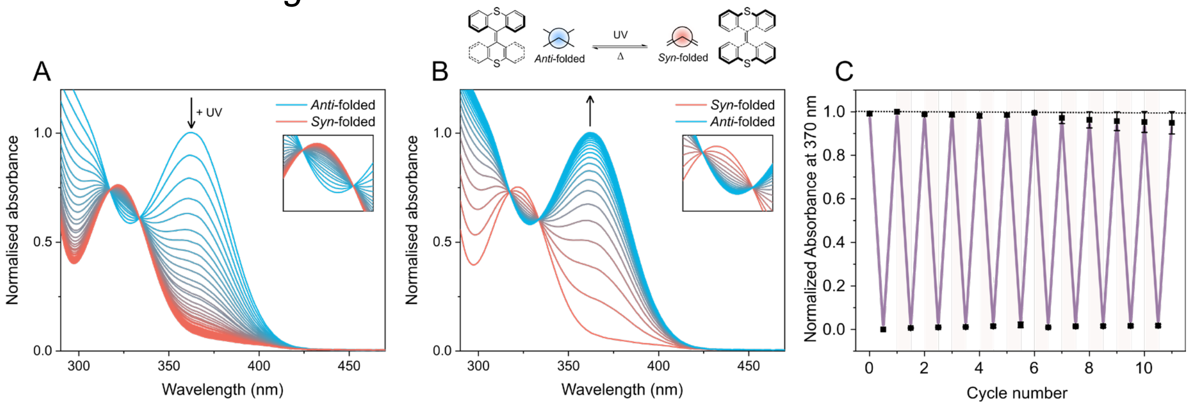

Supplementary Figure 8: Photo- and thermal switching of BTX-gel in water at 25 °C between *anti*- and *syn*-folded states as determined by UV/Vis spectroscopy. A) Changes in absorbance upon irradiation with 365 nm light. B) Changes in absorbance upon thermal relaxation. C) Photoswitching fatigue over 11 cycles followed by monitoring the absorbance at 370 nm. Error bars represent the standard deviation of two independent measurements.

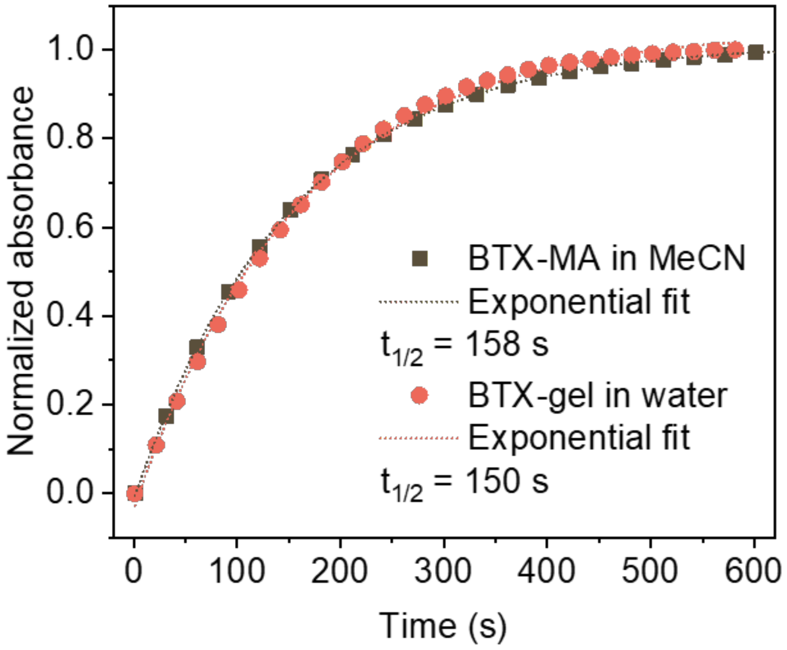

Supplementary Figure 9: Absorbance changes at 370 nm at 25 °C of the thermal relaxation of the *syn*-folded state to the *anti*-folded state for **BTX-gel** in water and **BTX-MA** in MeCN.

3.2 Oxidation & Reduction

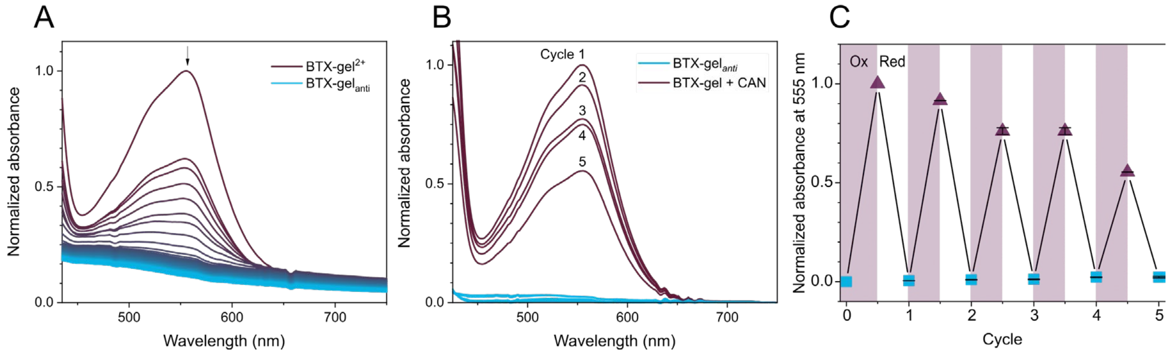

Supplementary Figure 10: A) UV/Vis spectra of **BTX-gel^{2+}** in a 15 mM solution of ascorbic acid, leading to reduction of **BTX-gel^{2+}** to neutral **BTX-gel**. Initially the *syn*-folded state is quantitatively formed which then thermally relaxes to the *anti*-folded state; in the depicted wavelength region neither of these species display any absorbance and due to the time scale of the experiment **BTX-gel$_{anti}$** will be the predominant species. B) Spectra showing the characteristic peak of **BTX-gel^{2+}** over five cycles of alternating oxidation and reduction. C) The normalized absorbance at 555 nm from B). Error bars represent one standard deviation of independent duplicate experiments. Note that the error bars are so small that they are barely visible.

It is important to note that the slow fatigue of bulk redox switching (Supplementary Figure 10C) is most likely related to a small degree of side reactions during/after oxidation/reduction and not due to incomplete redox switching. Nevertheless, even though there is some loss of activity, the absorbance peak at 555 nm still has an optical density of 1 after five cycles, and visually there is only a small decrease in the intensity of the color of the material.

4 Electrochemistry, Redox Mechanisms and Theoretical Calculations

4.1 General Electrochemical Characterization

As shown in Figure S11, the voltammetric properties of the monomer **BTX-MA** are largely unchanged over a large range of scan rates. The excellent linear dependence of the peak currents on the square-root of the scan rate further confirms that this redox process is diffusion-controlled (Supplementary Fig. 12).

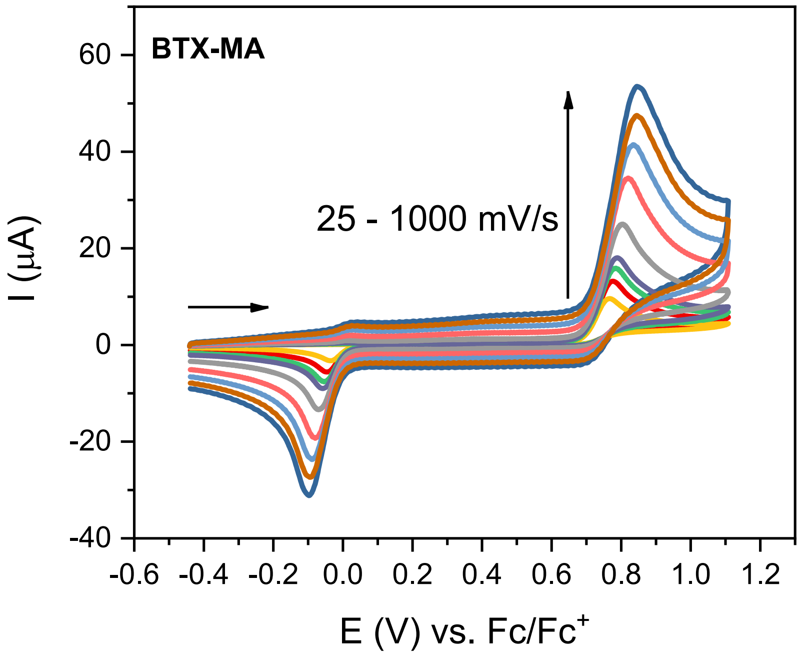

Supplementary Figure 11: CVs of 0.5 mM **BTX-MA** in CH_2Cl_2, 100 mM $TBAPF_6$ at varying scan rates.

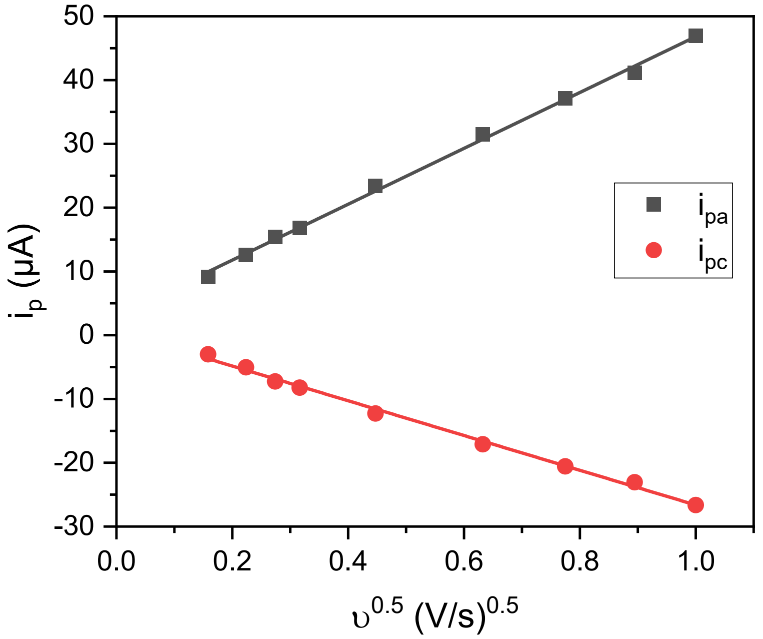

Supplementary Figure 12: Anodic and cathodic peak currents of 0.5 mM **BTX-MA** in CH_2Cl_2, 100 mM $TBAPF_6$ as a function of the square-root of the scan rate including linear fits.

Shown in Figure S13 is a comparison of the CVs of the *anti*- and *syn*-folded states of **BTX-MA** using different working electrodes (GC or Pt). As shown in Figure 3E in the main text, a very significant cathodic voltammetric shift was observed when using GC (~400 mV), however the oxidation peak for the *syn*-folded state is very broad under these conditions. In contrast, when using a Pt working electrode, a better-defined oxidation peak shape was obtained, however with a smaller cathodic shift of ~140 mV.

In any case, the potential shift is clearly large enough to enable selective, light-gated redox switching with Fe^{3+} in water.

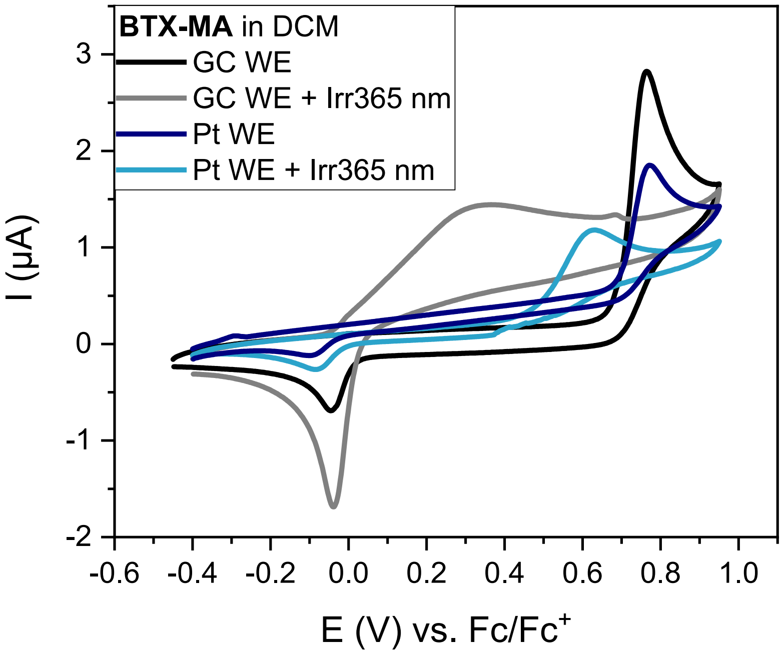

Supplementary Figure 13: CVs of 0.25 mM **BTX-MA** in CH_2Cl_2, 100 mM $TBAPF_6$ at v = 25 mV/s in both the *anti*- and *syn*-folded states. The *syn*-folded state was generated by continuous, in situ irradiation of the solution with 365 nm light. The CVs in blue were measured with a Pt disk working electrodes while the CVs shown in black were measured using a GC disk electrode.

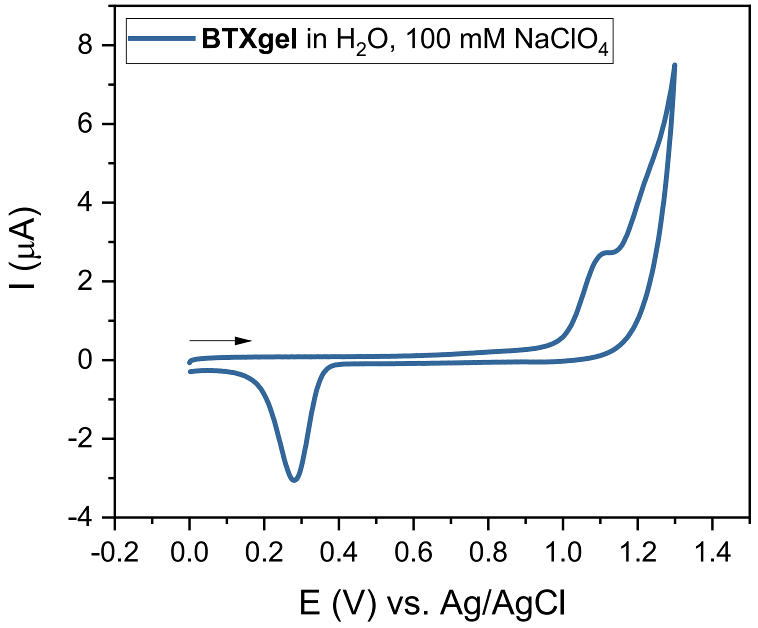

Supplementary Figure 14: CV of **BTX-gel** on ITO in H_2O, 100 mM $NaClO_4$ at v = 25 mV/s.

4.2 Mechanism of Redox Switching

Starting from the neutral, *anti*-folded state, one electron oxidation occurs at a high potential ($E_{Af,1}$) and transiently generates a radical cation in the same conformational state (*Af*$^{+\bullet}$, Figure S15). This species very quickly chemically rearranges to its most preferred conformational state (*Tw*$^{+\bullet}$, via k_1). This twisted radical cation now possesses a *lower* oxidation potential for removal of the second electron ($E_{Tw,2}$, with $E_{Tw,2} \ll E_{Af,1}$, i.e. potential inversion), such that the dicationic species in the twisted/orthogonal state (*Ort*$^{2+}$) is immediately generated (any redox potential sufficient to induce the first $E_{Af,1}$ process will automatically force the second electron transfer, such that both electrons are in effect transferred simultaneously and the intermediate radical cation *Tw*$^{+\bullet}$ is not formed in any significant amounts, giving rise to a simultaneous two-electron oxidation at ~0.8 V, see Figure 3 main text). In the forward direction (*anti*-folded → dication) the redox switching thus occurs via an ECE mechanism with a chemically irreversible first step, "ratcheted" by the favourable and fast geometric rearrangement of *Af*$^{+\bullet}$ to *Tw*$^{+\bullet}$). This was further supported by calculations of the relative conformer energies at the r^{2}SCAN-3c level of theory, which confirm that for the radical cation charge state, the *Af*$^{+\bullet}$ conformer is ~30 kJ/mol higher in energy than *Tw*$^{+\bullet}$ (see Table S1), such that the first one-electron transfer is (rapidly) followed by a chemical rearrangement step.

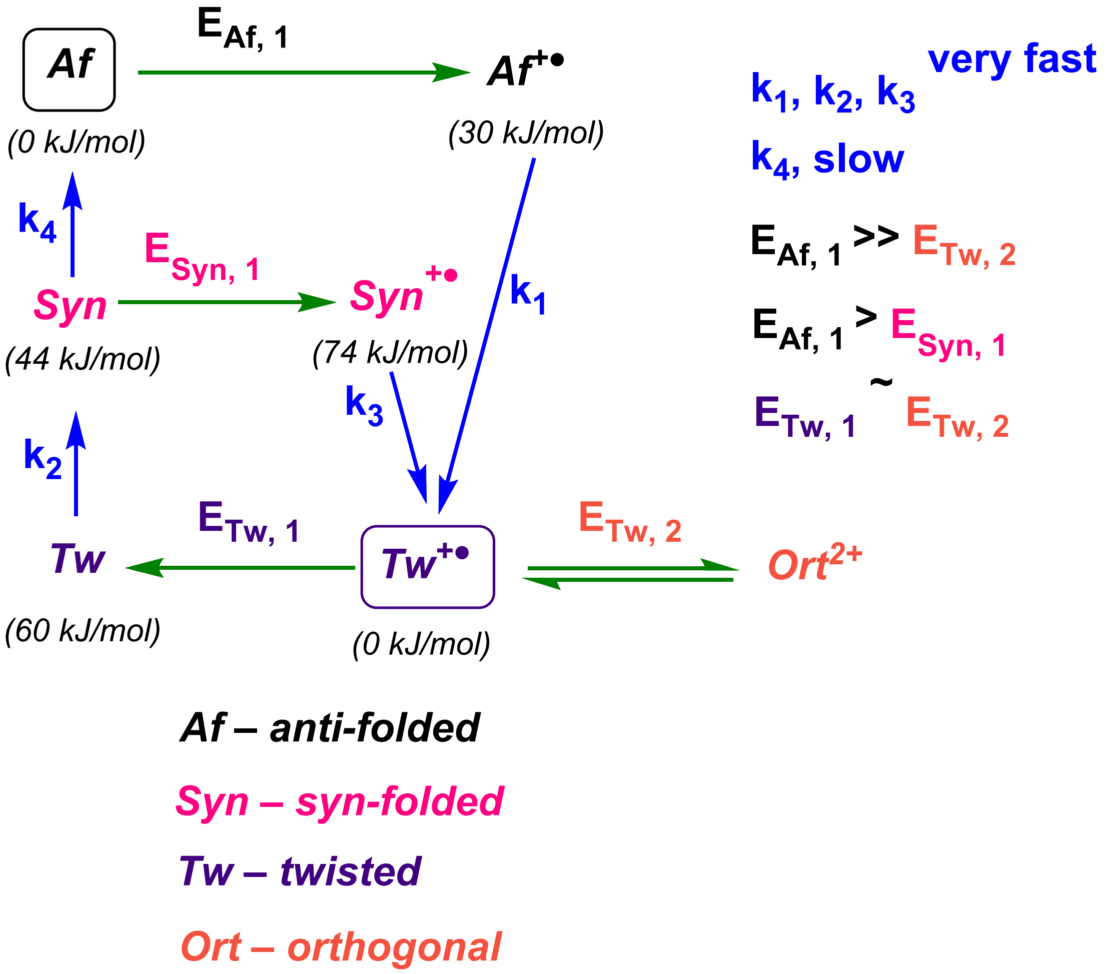

Supplementary Figure 15: Detailed square scheme depicting the relevant redox (green arrows) and structural (blue arrows) interconversions between all relevant redox states. The thermodynamically most stable and most relevant species for each oxidation state are highlighted with a box and for the neutral and radical cationic charge states the relative energies of all conformers are given in

parentheses (energy differences are given relative to the lowest lying conformer of each redox state, at 25 °C (vide infra). For simplicity, most pathways are depicted as only proceeding in one direction; however, in principle, all redox and structural processes are (reversible) equilibria; most depicted ET processes are rendered chemically irreversible through fast, subsequent rearrangements.

In principle, the second electron transfer between $Tw^{+\bullet}$ and Ort^{2+} is a fully reversible step as it occurs without any significant geometric rearrangements, or at least with very low barriers (Note that while for the BTX^{2+} dication, a perpendicular arrangement of the rotors has been confirmed crystallographically,[2] other more "twisted" conformations are energetically easily accessible by rotation around the central single-bond; in fact for a related derivative we recently chrystallographically observed a 64° angle between the mean planes of the two rotors in the dicationic "orthogonal" state).[3]

In spite of this reversible interconversion, the $Tw^{+\bullet}$ state can still not be accessed by reduction of the dication (at least not for the homomerous BTX derivatives[3]). This is because at potentials at which the reduction can be achieved (~ −0.05 V, see Figure 3 main text), the reduction of the second thioxanthylium rotor also occurs as there is very little electronic communication between the two halves in the single-bonded orthogonal state (i.e. $E_{Tw,1}$ ~ $E_{Tw,2}$, potential compression). As a result, during reduction simultaneous transfer of two electrons occurs easily, which transiently generates the neutral twisted state (*Tw*), which is an unfavored conformation for this charge state and thus very quickly rearranges to the *syn*-folded state (k_2). This was again confirmed by DFT calculations; for the neutral switch, *Tw* (~60 kJ/mol) is much higher in energy than the *syn*- (~44 kJ/mol) or *anti*-folded states (≡0 kJ/mol). The exclusive formation of *Syn* by reduction of Ort^{2+} (via the very unstable *Tw*) was confirmed previously.[2,4] As discussed in the main text, *Syn* can then thermally relax to *Anti* (k_4), which is however much slower than the other chemical rearrangements ($t_{1/2}$ ~ 150 s). As a result *Syn* can be quantitatively populated by irradiation and also oxidized, most likely via and ECE mechanism, this time via the $E_{Syn,1}$ and k_3 pathway and the transient generation of the $Syn^{+\bullet}$ state, whose relative energy is with 74 kJ/mol even higher than that of $Af^{+\bullet}$ (~30 kJ/mol). It is important to note that, despite the chemical irreversibility of the EC and/or EEC steps, the overall redox process displays a very high degree of practical reversibility, i.e. repeat interconversion of the neutral and dicationic states without significant decomposition/formation of other side-products.

The calculated structures and energies of all the relevant conformational and charge states discussed above are shown in Table S1 and Figures S16-27. For simplicity these calculations, as well as the following electrochemical studies were carried out using the 2,2`-dimethoxy-BTX precursor **1** as a simpler analogue of the BTX-MA monomer. Substitution at one of the methoxy groups to give the monomer is not expected to affect the switching properties of the core, even more significant substitutions do not significantly alter the general (redox) switching properties of the system.[1,4]

As shown in Table S1, the relative energies of the 2,2`-dimethoxy-BTX precursor **1** were calculated for both the *E* and *Z* isomers of all conformers and both the neutral and monocationic charge state. In all cases the energy difference between the *E* and

Z isomers is negligible, confirming that their (redox) switching behavior is the same, regardless which isomer is used (note we herein always use a mixture of both isomers).

Supplementary Table 1: Calculated Gibbs free energy differences (kJ/mol) at 25 °C of the different isomers of **1**, relative to the energy of the lowest isomer within each redox state. Calculations were performed at the r^{2}SCAN-3c level of theory in the gas phase.

	**Neutral**	**Radical Cation**
E-*anti*-folded	**0**	29.5
E-*syn*-folded	43.5	74.1
E-twisted	59.0	**0**
Z-*anti*-folded	**0.6**	29.6
Z-*syn*-folded	44.5	74.6
Z-twisted	60.0	**0.1**

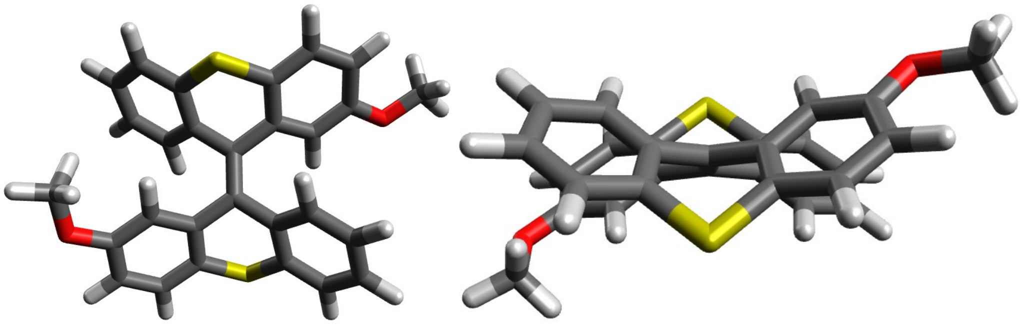

Supplementary Figure 16: Optimized geometry of the *E*-*anti* isomer of **1** (neutral state).

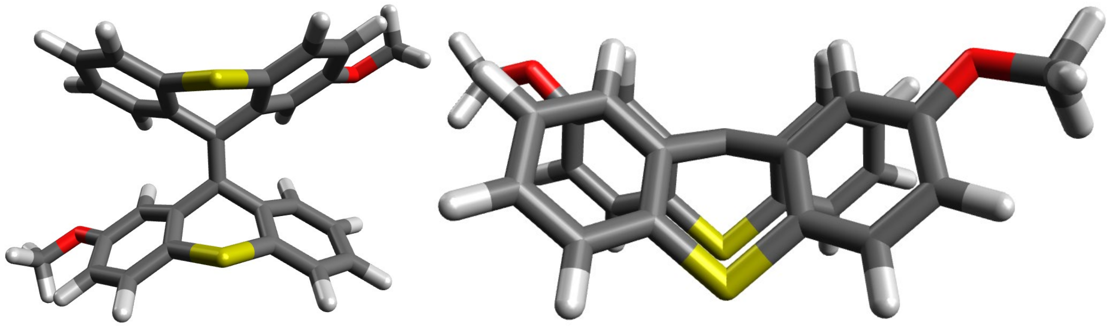

Supplementary Figure 17: Optimized geometry of the *E*-*syn* isomer of **1** (neutral state).

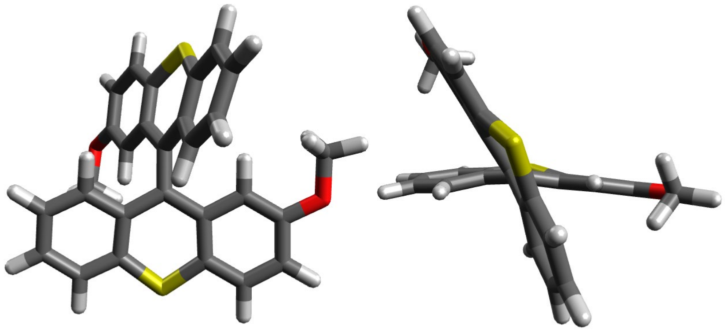

Supplementary Figure 18: Optimized geometry of the *E*-twisted isomer of **1** (neutral state).

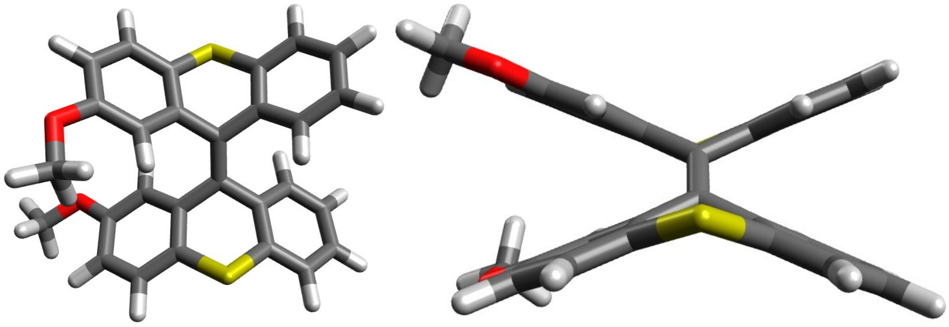

Supplementary Figure 19: Optimized geometry of the *Z-anti* isomer of **1** (neutral state).

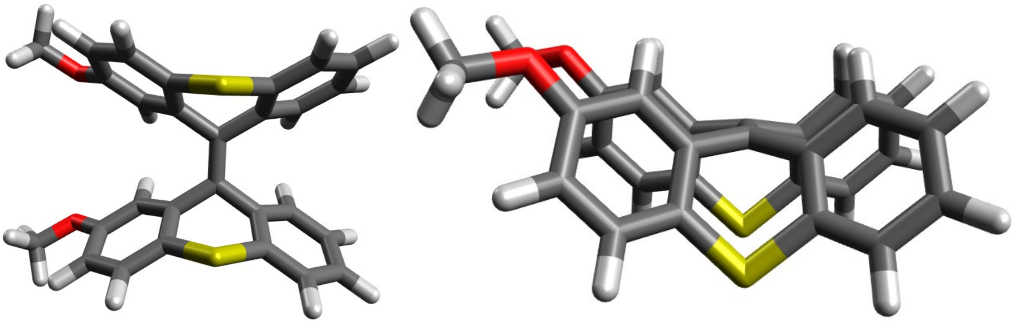

Supplementary Figure 20: Optimized geometry of the *Z-syn* isomer of **1** (neutral state).

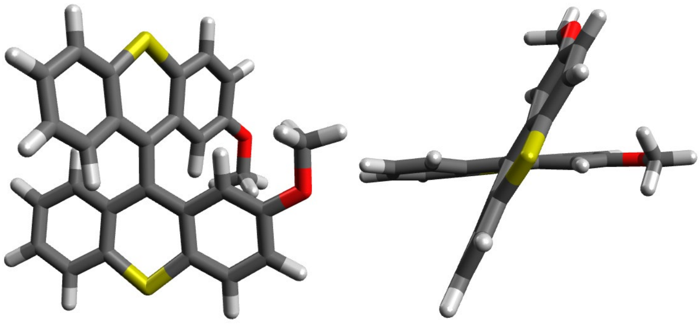

Supplementary Figure 21: Optimized geometry of the twisted *Z* isomer of **1** (neutral state).

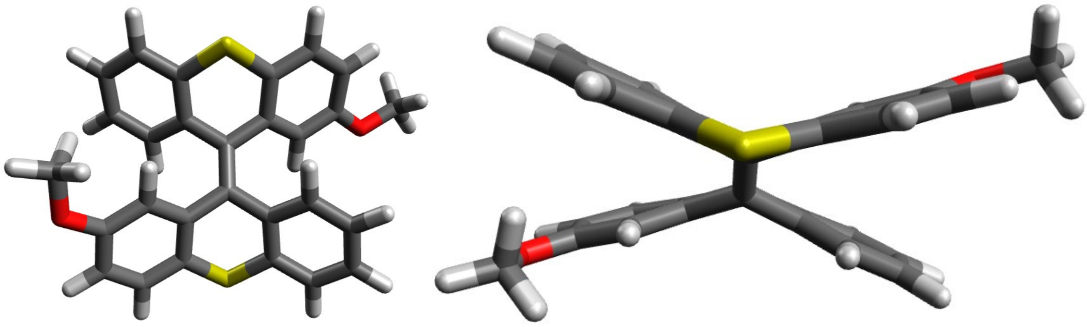

Supplementary Figure 22: Optimized geometry of the *E-anti* isomer of $\mathbf{1}^{\bullet+}$ (radical cationic state).

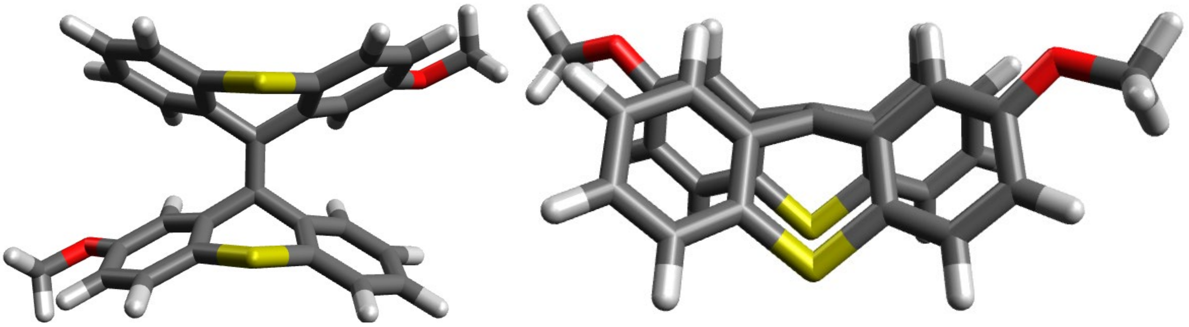

Supplementary Figure 23: Optimized geometry of the *E-syn* isomer of **1**$^{•+}$ (radical cationic state).

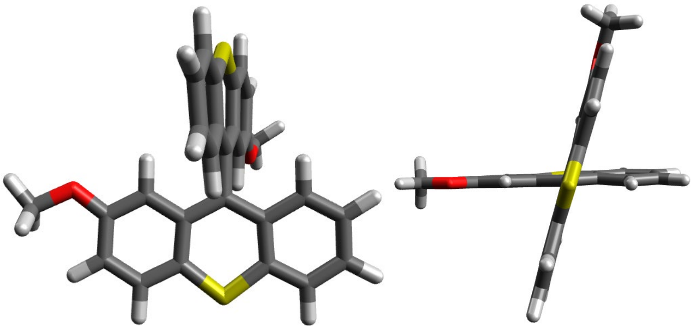

Supplementary Figure 24: Optimized geometry of the *E*-twisted isomer of **1**$^{•+}$ (radical cationic state).

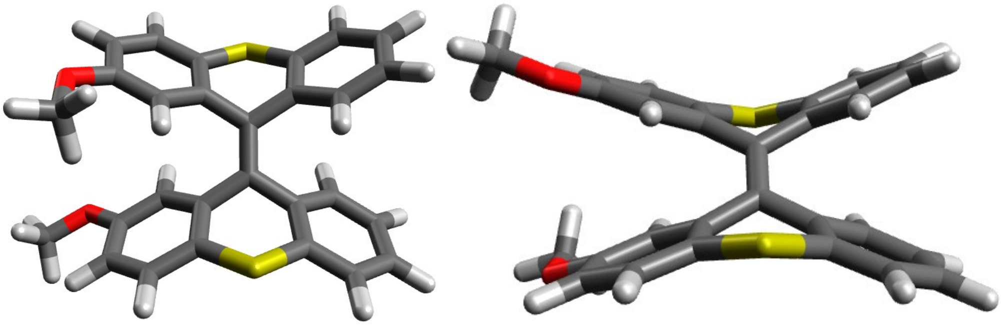

Supplementary Figure 25: Optimized geometry of the *Z-anti* isomer of **1**$^{•+}$ (radical cationic state).

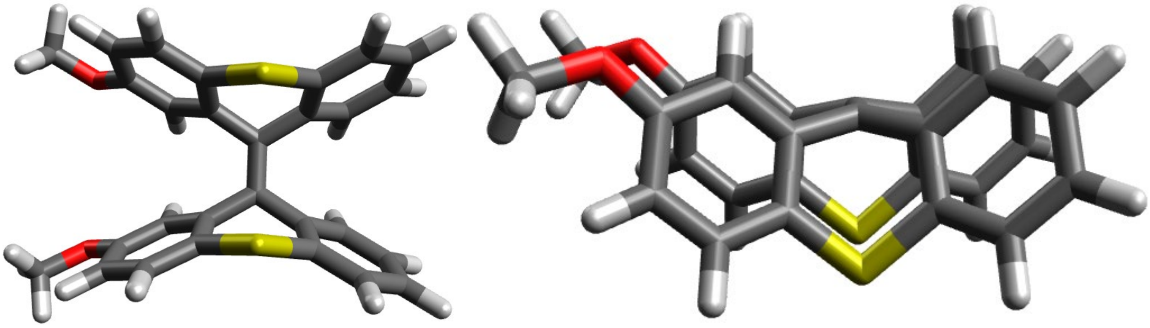

Supplementary Figure 26: Optimized geometry of the *Z-syn* isomer of **1**$^{•+}$ (radical cationic state).

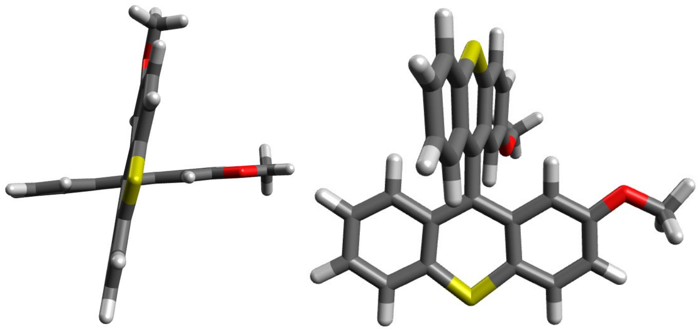

Supplementary Figure 27: Optimized geometry of the *Z*-twisted isomer of $\mathbf{1}^{\bullet+}$ (radical cationic state).

Number of transferred electrons

To further confirm that the single oxidative wave observed in the CVs corresponds to transfer of two electrons, we carried out the following additional experiments.

The time-dependent current response at a microdisk electrode following a potential step is given by the following equation as derived by Shoup and Szabo:[5]

$$I = 4nFDcrf(\tau) \qquad \textit{(eqn. S1)}$$

Where:

$$f(\tau) = 0.7854 + 0.8862 * \tau^{-0.5} + 0.214 * \exp\,[0.7823\tau^{-0.5}] \qquad \textit{(eqn. S2)}$$

and:

$$\tau = \frac{4Dt}{r^2} \qquad \textit{(eqn. S3)}$$

With *c* = bulk concentration, *n* = number of electrons transferred, *D* = diffusion constant, *r* = radius of the microdisk electrode and *F* = Faraday constant.

As shown by Compton and co-workers, both *D* and *n* can be simultaneously obtained by deconvolution of the current-response obtained from a simple chrono-amperometric experiment by non-linear curve fitting according to the equations above (when r and c are known).[6]
This analysis was carried out herein for **1** via chronoamperometry of a 1 mM solution of **1** in CH_2Cl_2, 100 mM $TBAPF_6$ using a Pt microdisk electrode (*r* = 5 µm) and OriginPro 2018. Figure S28 shows the CV under these conditions, while Figure S29 shows the CA current response.

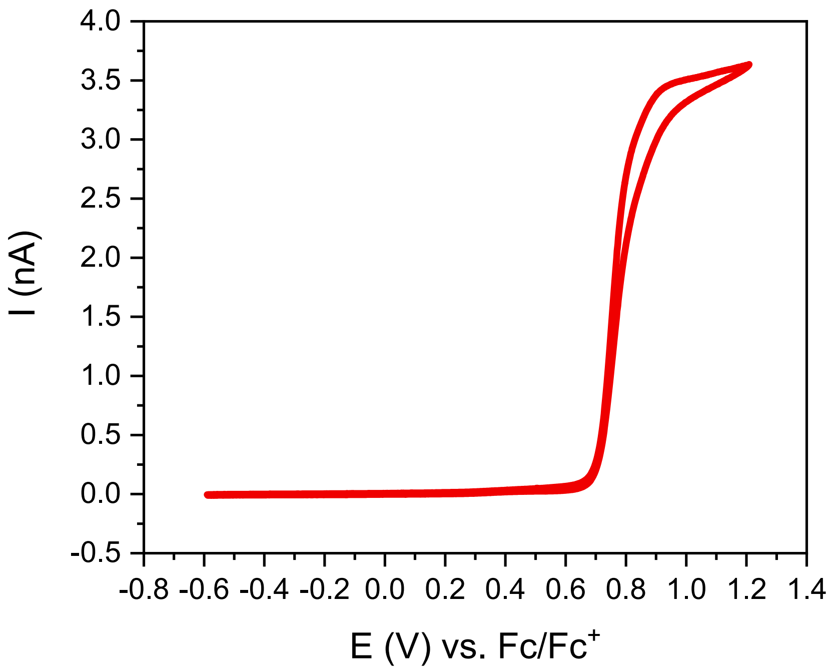

Supplementary Figure 28: CV of 1 mM **1** in CH_2Cl_2, 100 mM $TBAPF_6$ at a Pt micro disk electrode (r = 5 µm) at v = 100 mV/s.

Analysis of this current response according to the equations shown above affords a good fit to the experimental data with $D = 9.8 \times 10^{-10}$ m^2/s and n = 1.82, confirming that the oxidation wave of the BTX derivatives corresponds to transfer of two electrons.

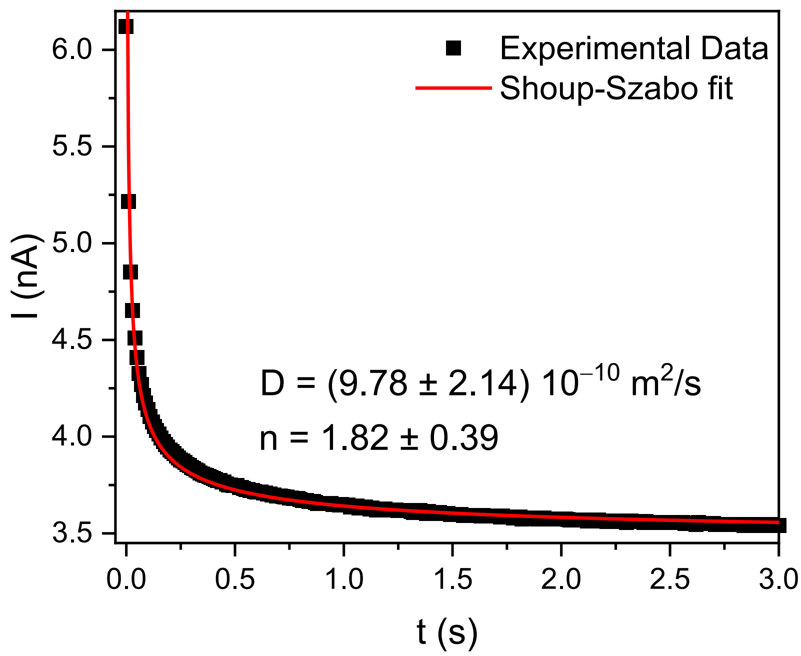

Supplementary Figure 29: Chronoamperometric current response (E = 1.01 V) for the oxidation of 1 mM **1** in CH_2Cl_2, 100 mM $TBAPF_6$ at a Pt micro disk electrode (r = 5 µm) including a fit according to the Shoup-Szabo equation.

This was further supported by controlled-potential bulk electrolysis of **1** in 1:1 (*v/v*) CH_3CN/CH_2Cl_2, 100 mM $TBAPF_6$ in a divided bulk electrolysis cell, which again confirmed that the oxidation wave is comprised of two electrons (Figure S30). Additionally, this confirmed that reduction also proceeds via two electrons. The mixed solvent system containing 50% CH_3CN was chosen to ensure good solubility of both the neutral and dicationic charge state of **1**.

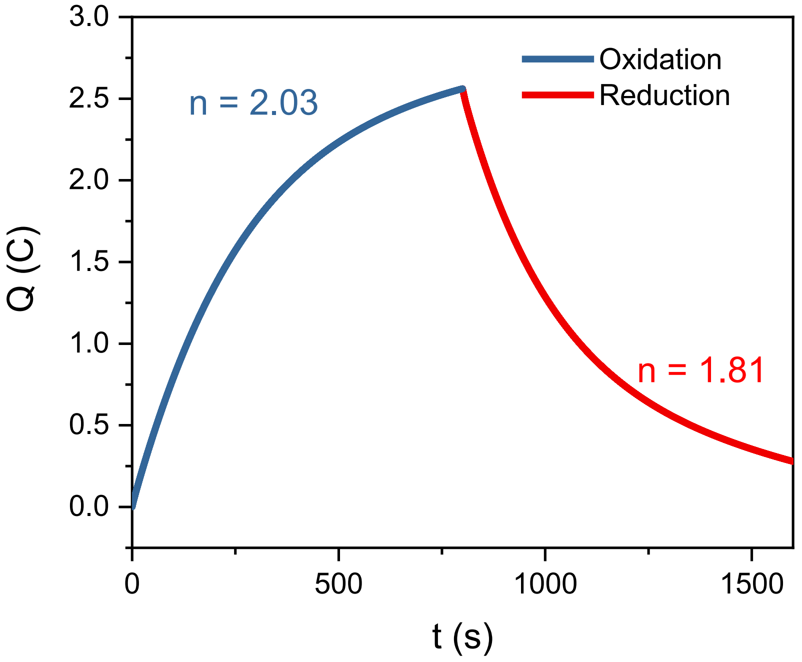

Supplementary Figure 30: Controlled-potential chronocoulometry of 13.1 µmol **1** in 1:1 (*v/v*) CH_3CN/CH_2Cl_2, 100 mM $TBAPF_6$ in a devided bulk electrolysis cell (WE: porous carbon, CE: Pt wire coil, RE: $Ag/AgNO_3$) confirming that both oxidation and reduction proceed via two electrons.

4.3 Mechanism for Change in Redox Potential Upon Irradiation

As mentioned in the main text, we hypothesized that the lowered oxidation potential of the *syn*-folded BTX switch state is related to its first ionization potential, i.e. its HOMO energy, for the first one electron oxidation ($E_{Syn,1}$ and $E_{Af,1}$ in Figure S15). According to Koopmans' theorem,[7] within the Hartree-Fock approximation, the first ionization energy of a molecule is equal to the negative of the energy of the HOMO. While this theorem is not exact for DFT-calculated energies, numerous studies report accurate predictions of ionization potentials using this method at the DFT level.[8,9]

Figures S31-32 and Table S2-3 show the valence orbitals energy diagram and highest occupied orbitals at the ωB97X-D4/def2-TZVPD level of theory for both folded isomers of *E*-**1** and *Z*-**1**, respectively. As shown in Figures S31 and S32, the HOMO energies for all isomers (*E* and *Z* as well as *syn*- and *anti*-folded) are very similar.

Specifically, at the ωB97X-D4/def2-TZVPD level of theory, the following difference in ionization potential (IP) are obtained between the *syn*- and *anti*- forms of the *E*- and *Z*-isomers:

$\Delta IP_{anti/syn}(E\text{-}\mathbf{1}) = IP_{anti}(E\text{-}\mathbf{1}) - IP_{syn}(E\text{-}\mathbf{1}) = -\varepsilon HOMO(E\text{-}anti\text{-}\mathbf{1}) - (-\varepsilon HOMO(E\text{-}syn\text{-}\mathbf{1})) = 7.7969 - 7.7901 = \mathbf{0.0068\ eV}$ *(eqn. S4)*

$\Delta IP_{anti/syn}(Z\text{-}\mathbf{1}) = IP_{anti}(Z\text{-}\mathbf{1}) - IP_{syn}(Z\text{-}\mathbf{1}) = -\varepsilon HOMO(Z\text{-}anti\text{-}\mathbf{1}) - (-\varepsilon HOMO(Z\text{-}syn\text{-}\mathbf{1})) = 7.7915 - 7.7751 = \mathbf{0.0164\ eV}$ *(eqn. S5)*

This suggests that the *syn*- form of both *E* and *Z* isomers are slightly easier to oxidize than the *anti*- form, with a difference of 7 and 16 mV respectively. However, these differences are too small to explain the experimentally observed difference in oxidation potential, whereby the syn-folded state displays at least 100 mV lower E_{Ox} (Figure S13). This suggests that other factors need to be considered to explain the full extent of the observed difference in oxidation potentials. Specifically, this could be related to the kinetics of the “C” step or a complete change in oxidation mechanism (e.g. CEE), however this requires further in-depth electrochemical and theoretical studies, which are beyond the scope of this work.

Lastly, we would also like to highlight that these calculations correctly predict an increase in HOMO-LUMO gap upon light-induced switching to the syn-folded state, as is indeed observed experimentally by a blue-shift in the absorbance spectrum (Figure 3B).

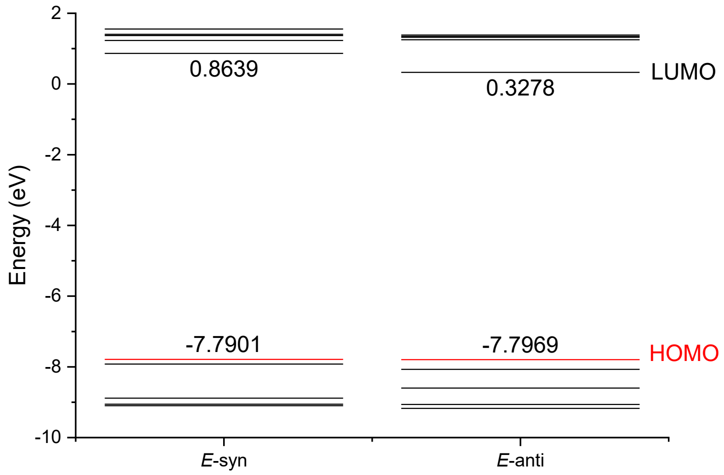

Supplementary Figure 31: Valence orbitals energy diagram (LUMO-4 to HOMO+4) for the *E-syn* and *E-anti* isomers of **1** at the ωB97X-D4/def2-TZVPD level of theory.

Supplementary Table 2: Plot of the highest occupied orbitals for the *E-syn* and *E-anti* isomers of **1** (ωB97X-D4/def2-TZVPD).

	E-syn	*E-anti*
HOMO	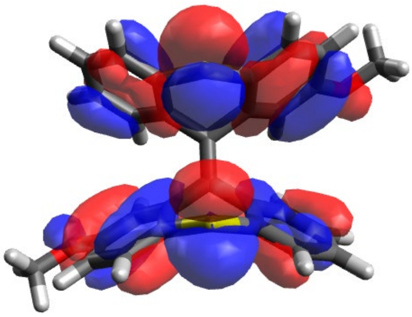	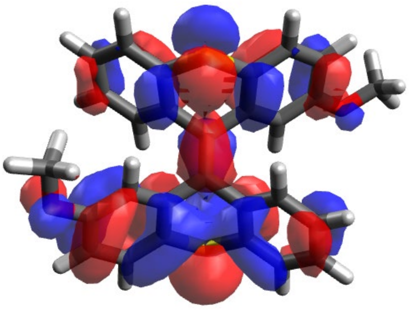
HOMO-1	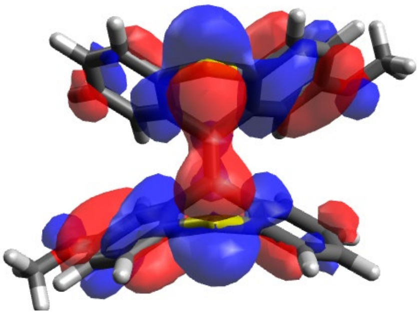	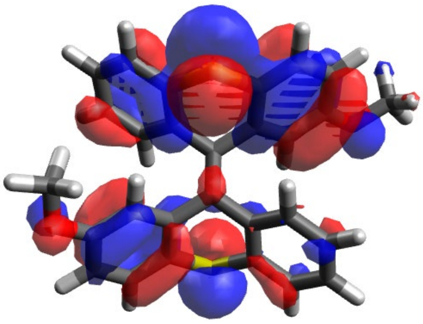
HOMO-2	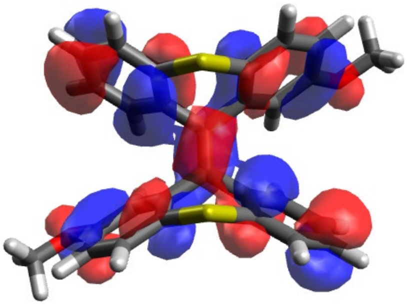	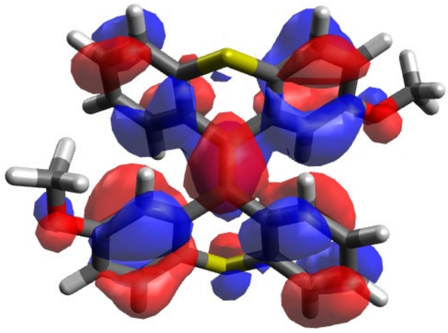
HOMO-3	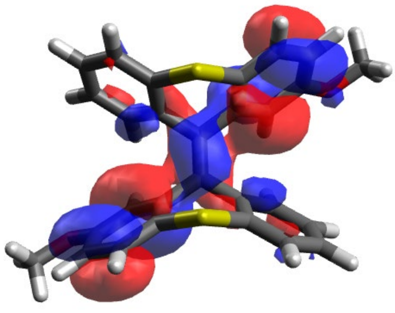	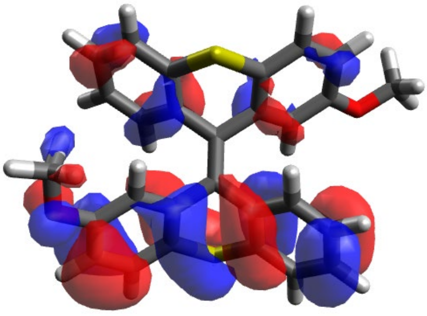

HOMO-4	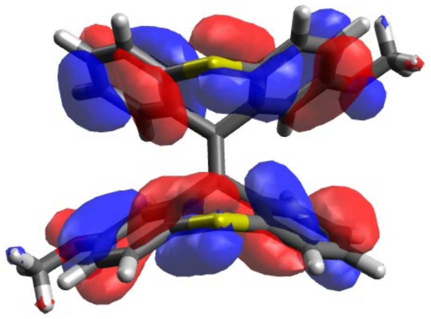	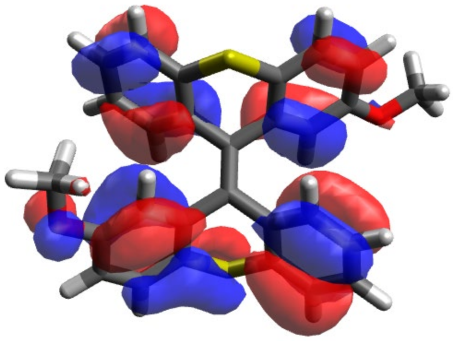

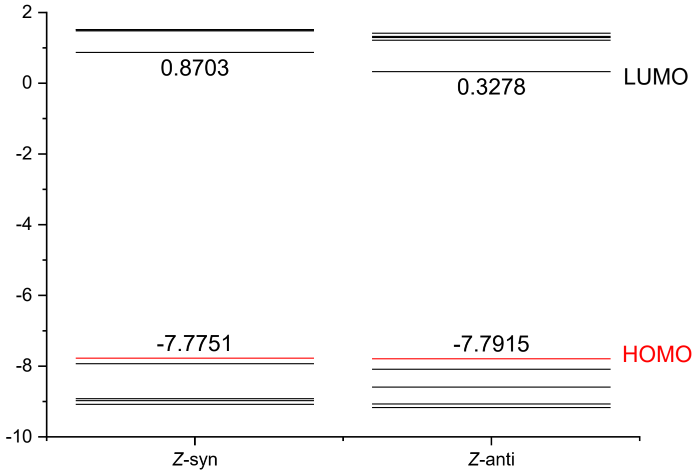

Supplementary Figure 32: Valence orbitals energy diagram (LUMO-4 to HOMO+4) for the *Z-syn* and *Z-anti* isomers of **1** at the ωB97X-D4/def2-TZVPD level of theory.

Supplementary Table 3: Plot of the highest occupied orbitals for the *Z-syn* and *Z-anti* isomers of **1** (ωB97X-D4/def2-TZVPD).

	Z-syn	*Z-anti*
HOMO	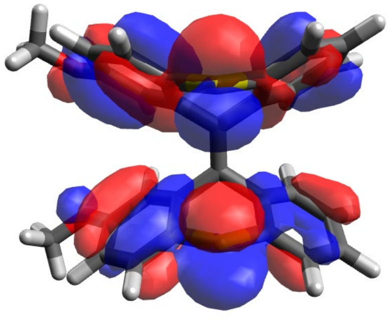	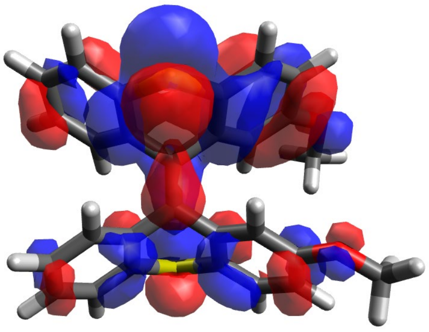

HOMO-1	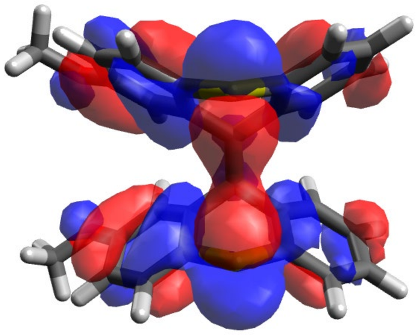	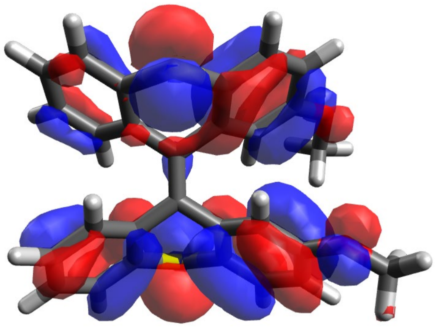
HOMO-2	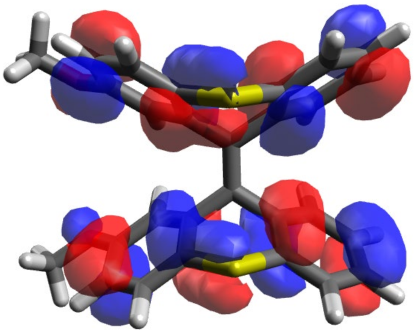	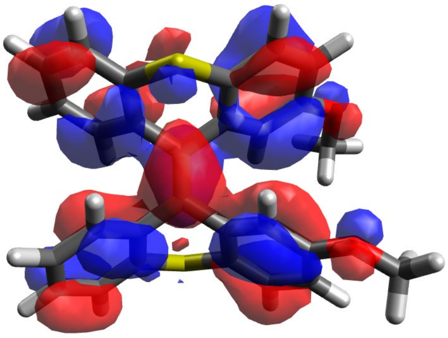
HOMO-3	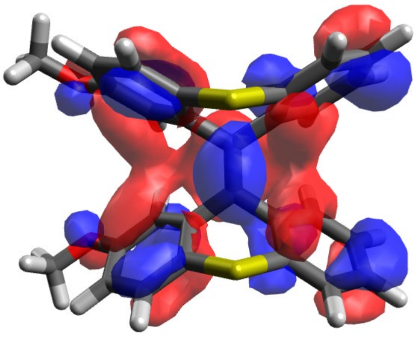	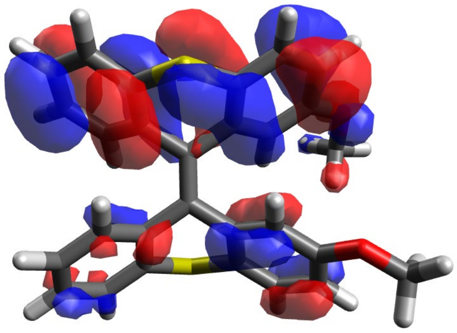
HOMO-4	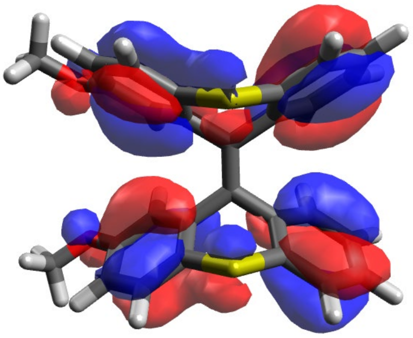	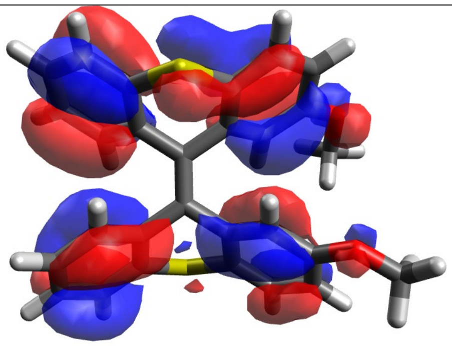

5 Material Optimization & Rheology

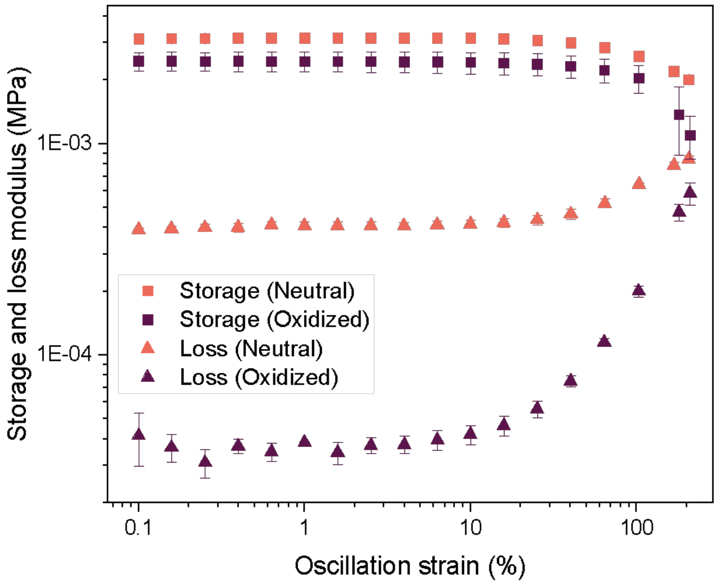

Supplementary Figure 33: Plot of the storage (*G'*) and loss modulus (*G''*) of **BTX-gel** and **BTX-gel^{2+}** vs. the oscillation strain. Error bars represent one standard deviation of independent triplicate experiments.

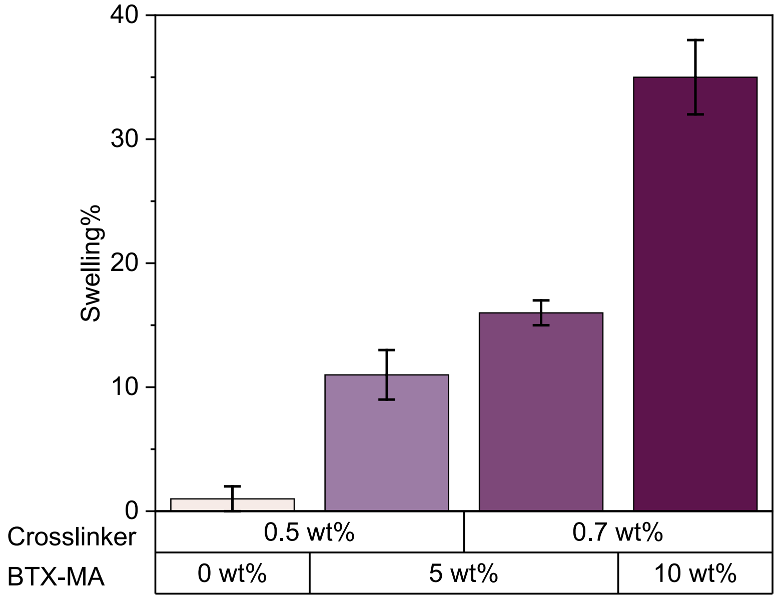

Supplementary Figure 34: Bar graph showing the oxidation-induced swelling for several different material compositions. Error bars represent one standard deviation of independent triplicate experiments.

6 Swelling

The effect of the oxidant strength (CAN or $Fe(ClO_4)_3$) and irradiation (dark or 365 nm light) on the swelling of the BTX hydrogels was studied and the results are summarized in Figure S40. For each condition, representative photographs of the hydrogel pieces are shown in Figures S36-39. To ensure that the swelling effect is due to BTX, a 'blank' gel consisting of the same components as the **BTX-gel** except **BTX-MA** was also treated with CAN. This did not lead to swelling, proving that the increase in size of the gels is due to oxidation from BTX to BTX^{2+} (Figure S35). Every experiment described below was repeated at least three times.

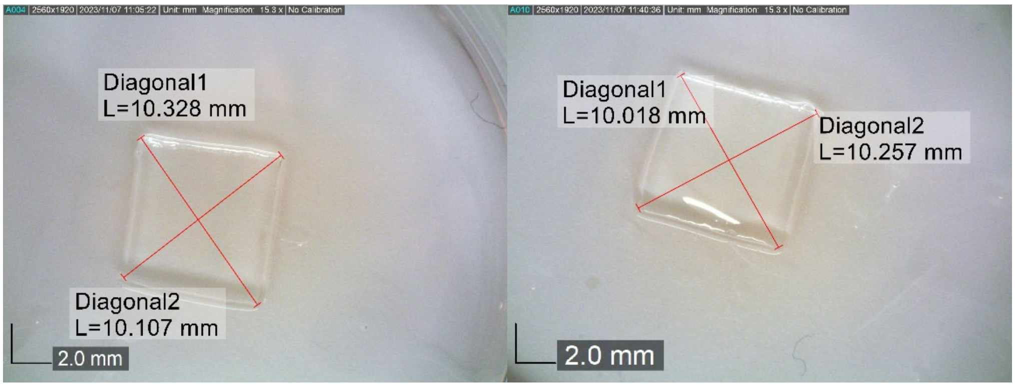

Supplementary Figure 35: Photographs of a piece of 'blank' gel consisting of 99% *N,N*-dimethylacrylamide, 0.7% *N,N*-methylenebisacrylamide and 0.3% IRG819, i.e. the polymer matrix without BTX, before (left) and after (right) treatment with 15 mM CAN. This experiment was performed to exclude any influence of CAN on the swelling of the polymer matrix without BTX. No change was observed in these gels after treatment with CAN.

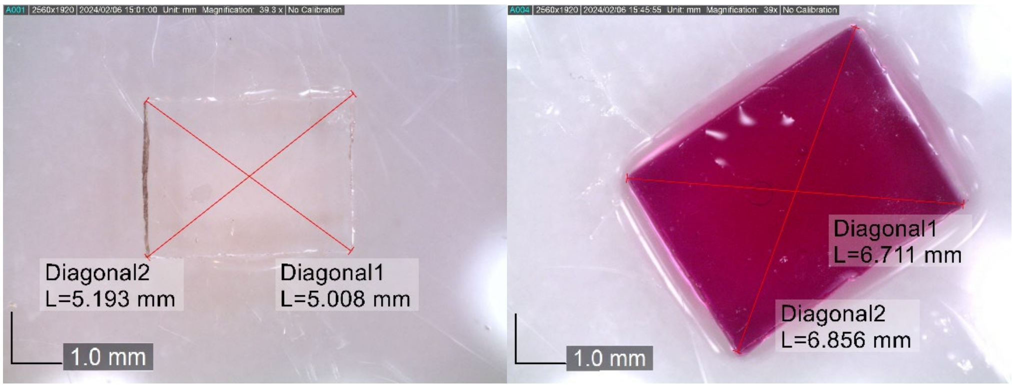

Supplementary Figure 36: Photographs of a piece of **BTX-gel** before (left) and after (right) treatment with 15 mM CAN in water.

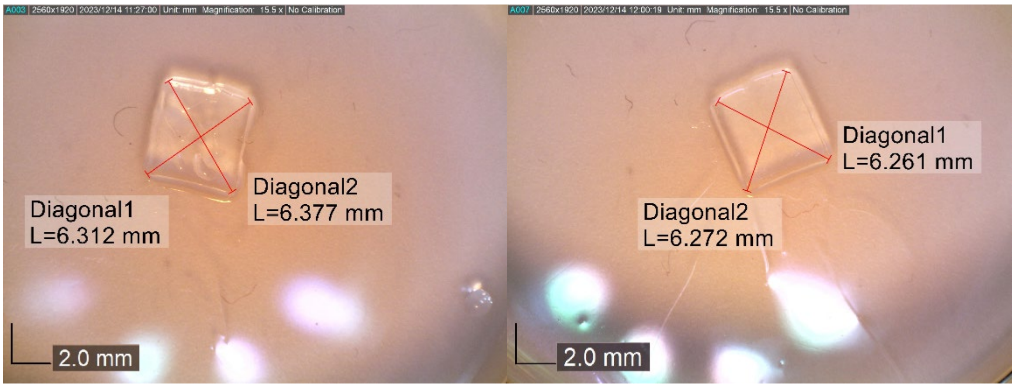

Supplementary Figure 37: Photographs of a piece of **BTX-gel** in water before (left) and after (right) treatment with 30 mM $Fe(ClO_4)_3$ without irradiation.

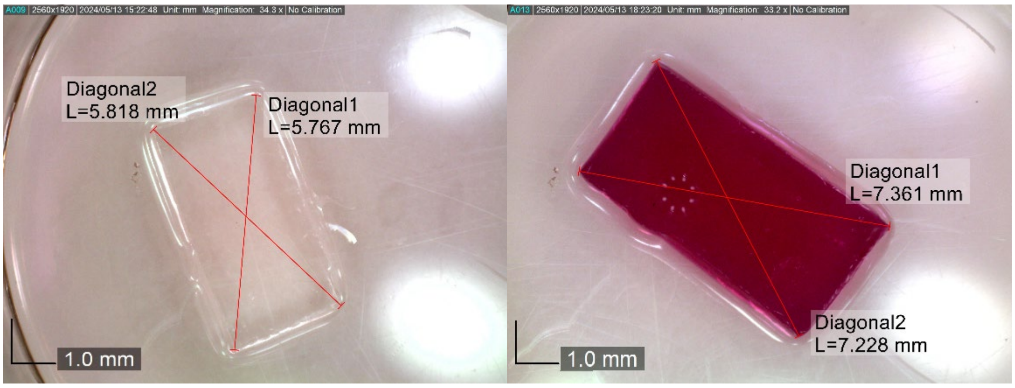

Supplementary Figure 38: Photographs of a piece of **BTX-gel** in water (left) and after treatment with 30 mM $Fe(ClO_4)_3$ and 365 nm light (right).

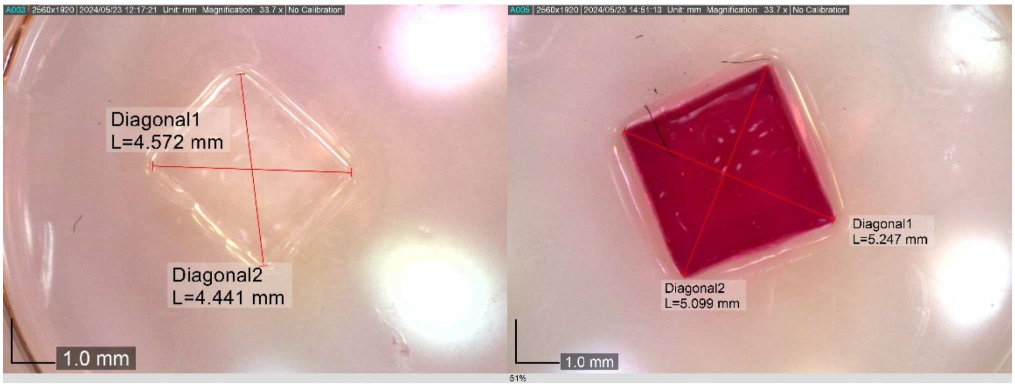

Supplementary Figure 39: Photographs of a piece of **BTX-gel** in water (left) and after treatment with 15 mM CAN and 365 nm light (right).

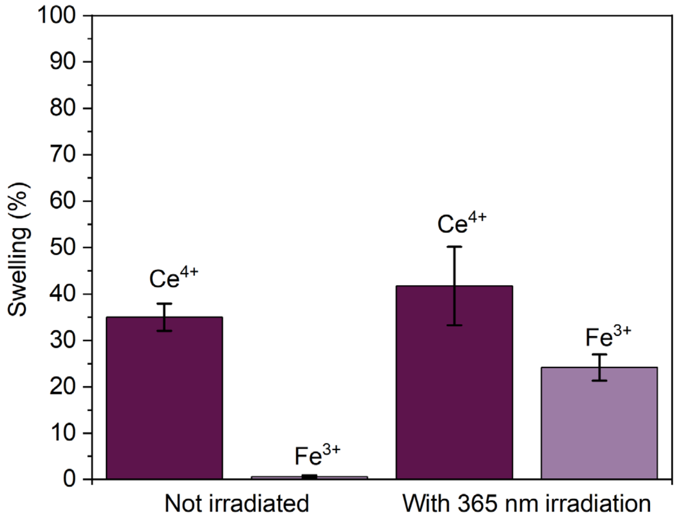

Supplementary Figure 40: Bar graph illustrating the %increase in characteristic length of **BTX-gel** squares in different oxidation conditions. Using CAN in the dark led to a 35 ± 3% increase in size, and $Fe(ClO_4)_3$ without irradiation had no effect on the size of the gel. CAN with 365 nm light irradiation caused the material to swell 42 ± 8% and $Fe(ClO_4)_3$ with 365 nm irradiation caused the material to swell 24 ± 3%. Error bars represent one standard deviation of independent triplicate experiments.

Swelling experiments in the presence of various salts and pH

In order to study the influence of various salt and pH conditions on the swelling behaviour of the neutral gel, as well as for the chemical oxidation with CAN, the following experiments were carried out:

The size of the **BTX-gel** was first measured in pure water. The medium was then exchanged with the salt solution and after 30 minutes of equilibration the size was measured again. Afterwards, CAN was added (15 mM total, added as a stock solution of same salt concentration/pH) and the oxidized gels were equilibrated in the oxidant for 2 hours. The oxidized gels were then washed with pure salt solution and their size remeasured.

As shown in Figure S41, the nature of the counter anion (NO_3^-, PF_6^- or ClO_4^-) did not significantly alter the swelling properties of the gel, neither in the neutral state, nor after oxidation. Only for PF_6^- a slightly smaller swelling magnitude was observed after oxidation, however this can in part be attributed to some degree of precipitation formed by reaction between the CAN oxidant and the counter anion. Nevertheless, gel oxidation was still observed.
As shown in Figures S42 and S43, increasing the ionic strength (either by increase of the electrolyte concentration ($NaNO_3$) or by lowering the pH using HNO_3 (at a constants background concentration of 100 mM $NaNO_3$) induced a small decrease in the degree of swelling upon oxidation. For the swelling of the neutral gels upon exchange of the medium from pure water to the electrolyte, no clear trends that correlate with overall ionic strength were observed, however the change in gel size was comparably small in all cases (max 5 ± 2%) for 100 mM $NaNO_3$ at pH 7.

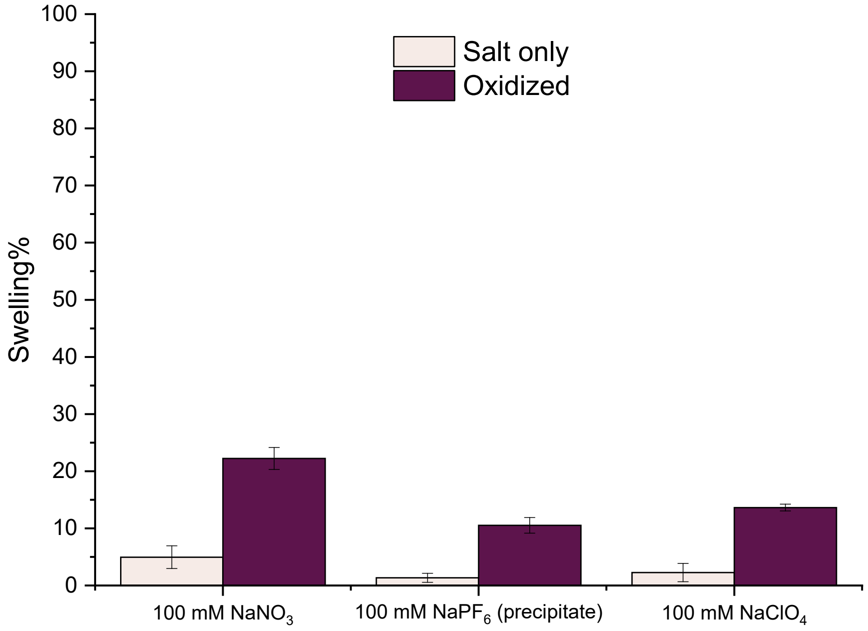

Supplementary Figure 41: Swelling percentage (% increase in the characteristic length) of **BTX-gel** after the exchange of the swelling medium from water to a 100 mM sodium salt solution (off-white bars) and after oxidation using CAN (15 mM) in the same salt solution (purple bars), relative to the size of the gel in water. Upon addition of CAN to the gel in 100 mM $NaPF_6$ some precipitate was observed, however the oxidation still took place. Error bars represent one standard deviation of independent triplicate experiments.

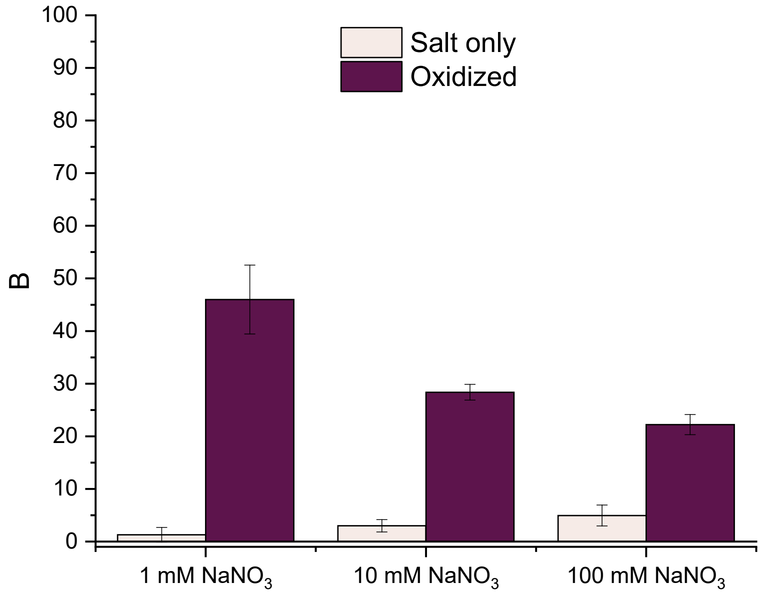

Supplementary Figure 42: Swelling percentage (% increase in the characteristic length) of **BTX-gel** after the exchange of the swelling medium from water to $NaNO_3$ solutions with different concentrations (off-white bars) and after oxidation using CAN (15 mM) in the same salt solution (purple bars), relative to the size of the gel in water. Error bars represent one standard deviation of independent triplicate experiments.

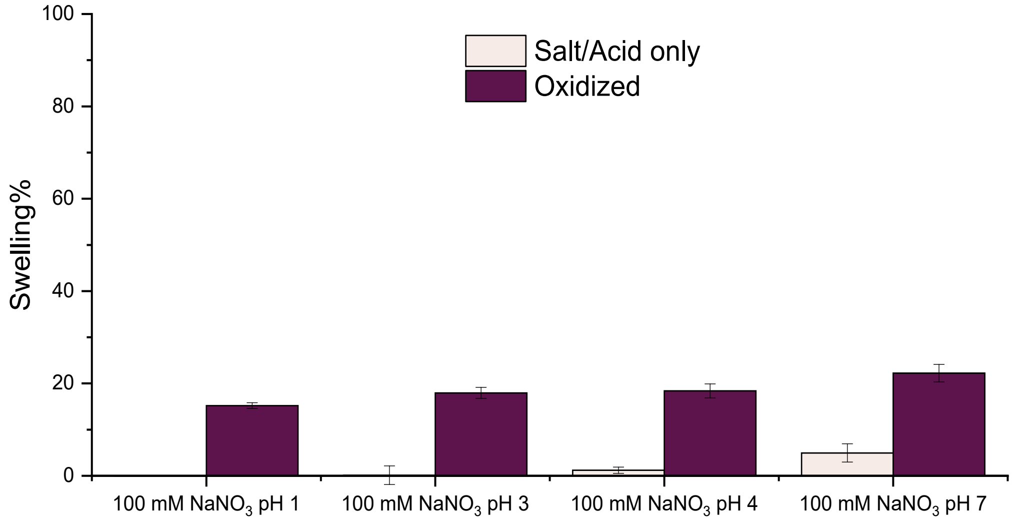

Supplementary Figure 43: Swelling percentage (% increase in the characteristic length) of **BTX-gel** after the exchange of the swelling medium from water to a 100 mM $NaNO_3$ solution with different pH (off-white bars) and after oxidation using CAN (15 mM, at the same pH; purple bars), relative to the size of the gel in water. The pH was adjusted using HNO_3. Error bars represent one standard deviation of independent triplicate experiments.

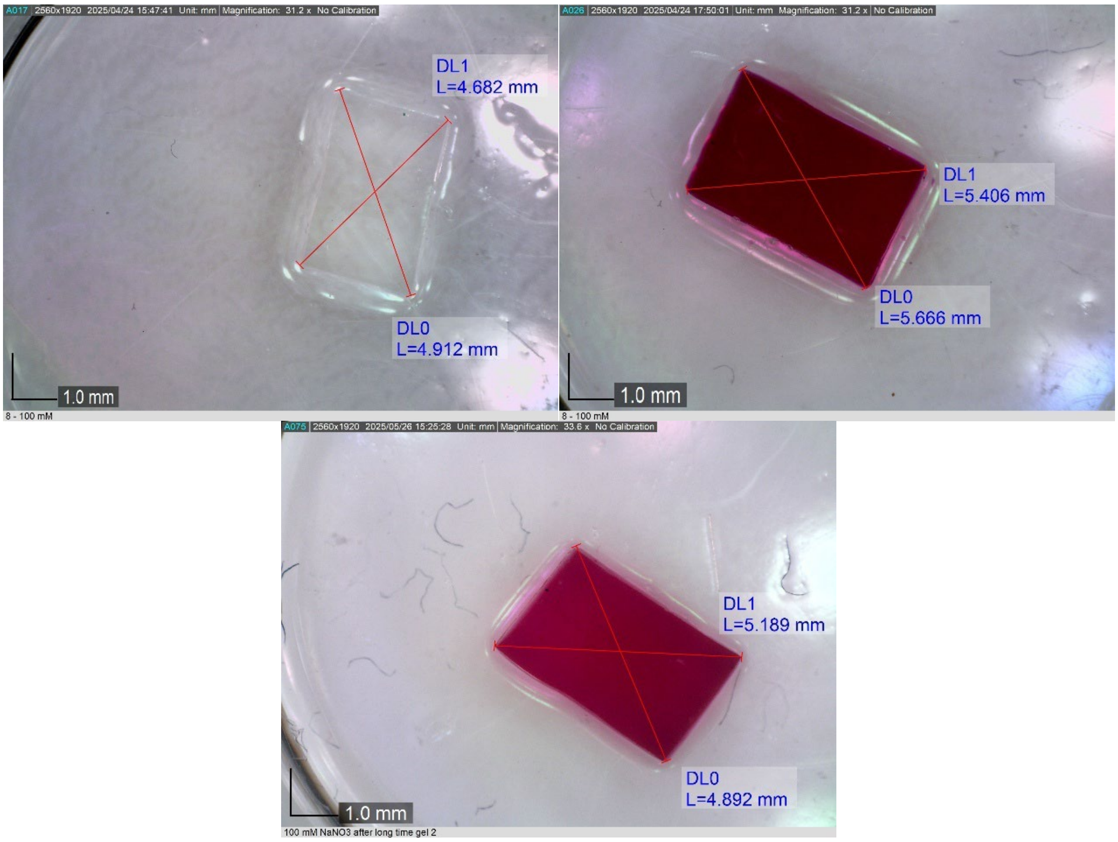

Supplementary Figure 44: Pictures of the BTX-gel in 100 mM $NaNO_3$ (top left), after oxidation with 15 mM CAN (top right) and after one month in the salt solution (due to some solvent evaporation the exact concentration of $NaNO_3$ is not known).

7 Actuation

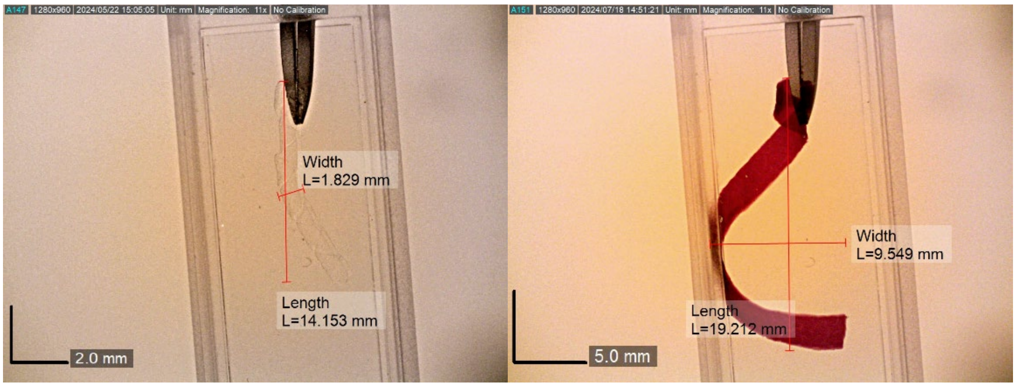

Supplementary Figure 45: BTX-gel helix prepared *via* Method 2 before (left) and after (right) oxidation with CAN.

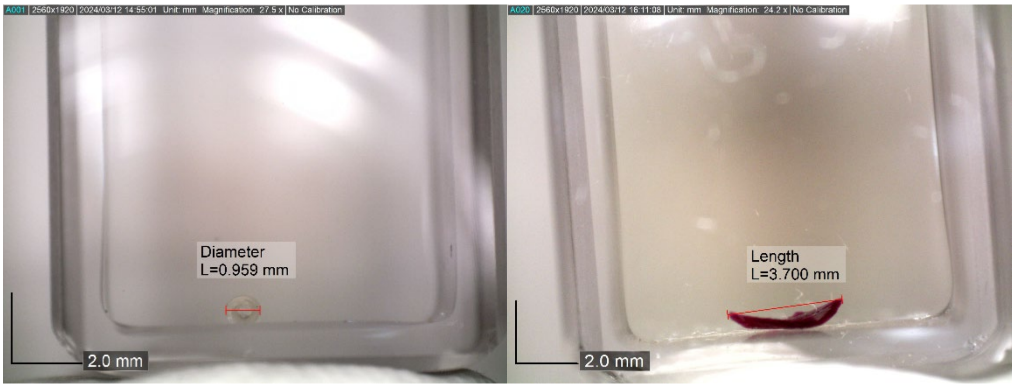

Supplementary Figure 46: BTX-gel curl prepared *via* Method 2 before (left) and after (right) oxidation with CAN.

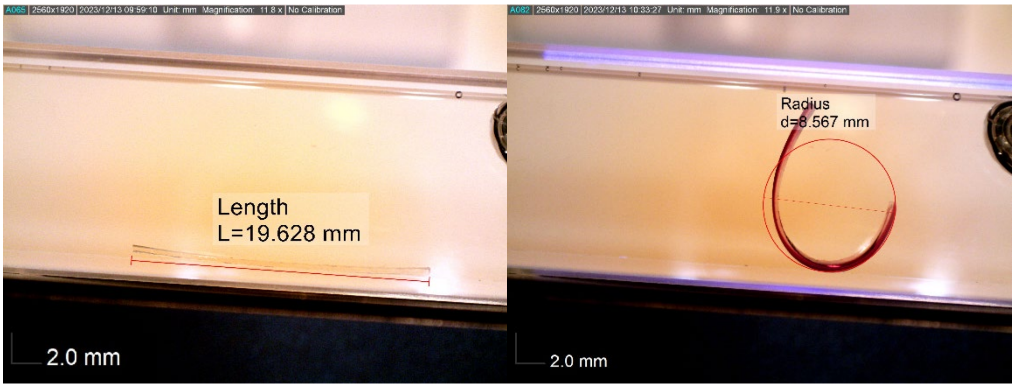

Supplementary Figure 47: Isotropic **BTX-gel** strip in 30 mM $Fe(ClO_4)_3$ prepared via Method 1 before (left) and after (right) continuous 365 nm irradiation from the bottom.

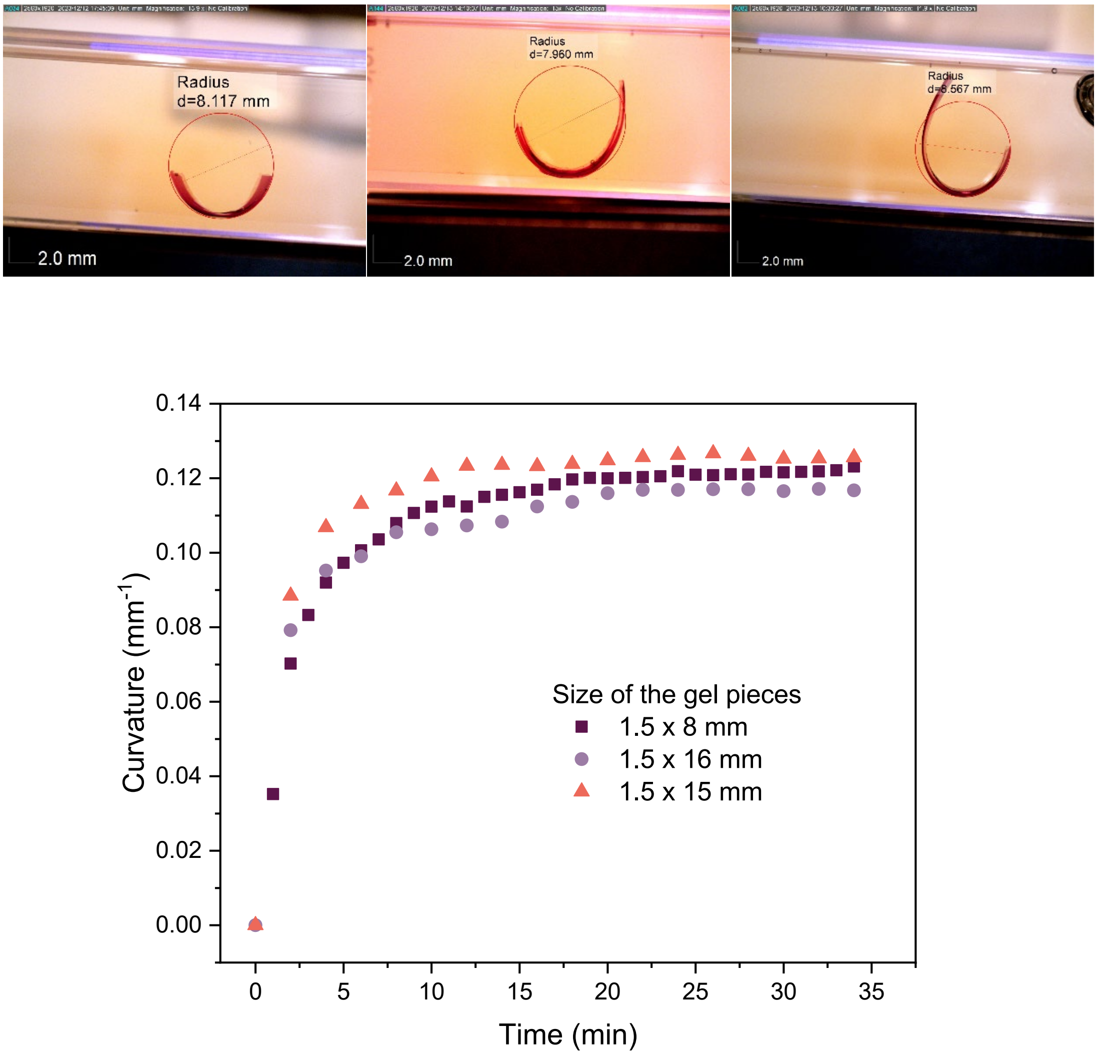

Supplementary Figure 48: (TOP) Images of three isotropic **BTX-gel** strips in 30 mM $Fe(ClO_4)_3$ prepared via Method 1 after irradiation and 365 nm irradiation from the bottom. (BOTTOM) Plot of the change in curvature (1/r, r = radius) over time of these three isotropic **BTX-gel** strips in 30 mM $Fe(ClO_4)_3$ prepared under continuous 365 nm irradiation from the bottom.

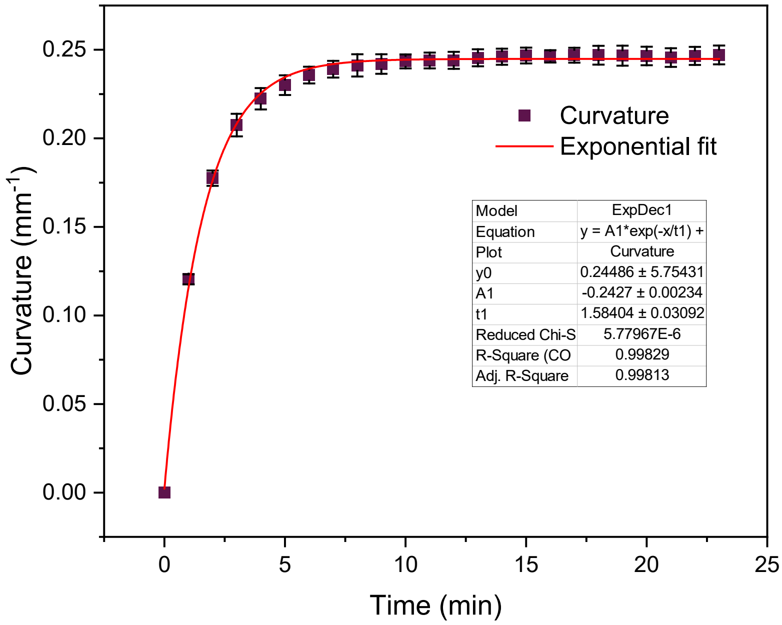

Supplementary Figure 49: Curvature of the gel strips over time under continuous 365 nm irradiation in the presence of 30 mM $Fe(ClO_4)_3$. Error bars represent one standard deviation of three independent samples sized 2 x 14 mm.

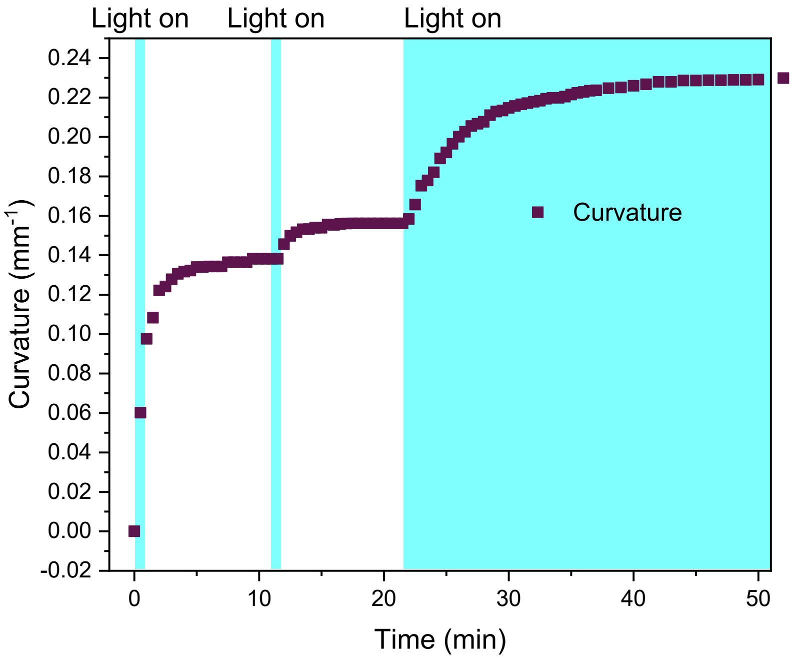

Supplementary Figure 50: Curvature of a gel strip over time with intermittent 365 nm irradiation (cyan) and in the dark (white) in the presence of 30 mM $Fe(ClO_4)_3$. The first two shorter irradiations were 20 s each.

8 Fluorescence

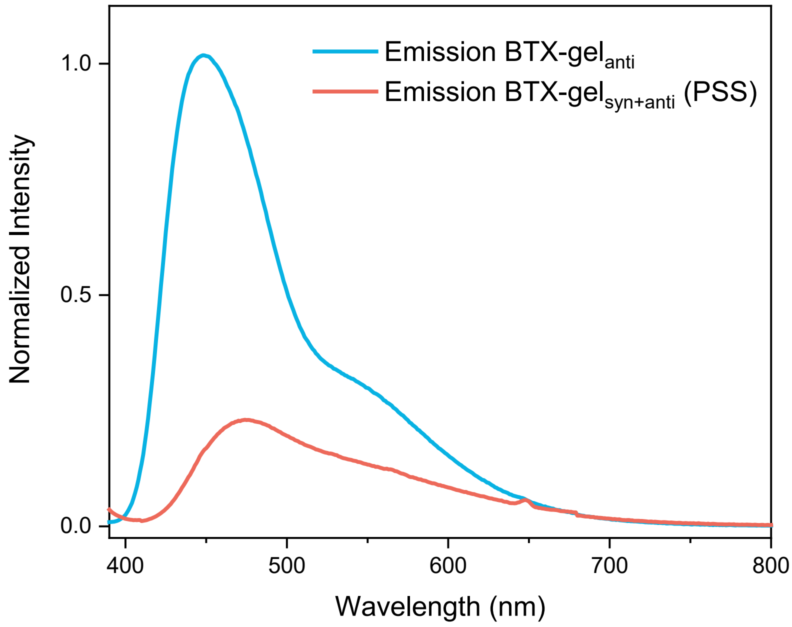

Supplementary Figure 51: Normalized fluorescence emission spectra (λ_{ex} = 365 nm) of **BTX-gel*anti*** in water and after 5 min ex-situ irradiation with 365 nm light, corresponding to mostly **BTX-gel*syn*** There is some residual fluorescence because it was difficult to fully convert every *anti*-folded BTX into *syn*-folded inside the hydrogel. Additionally, it was not possible to irradiate with the 365 nm LED while recording the emission spectrum. Therefore, considering the half-life of *syn*-folded BTX, there is always going to be a small amount of relaxation during the measurement.

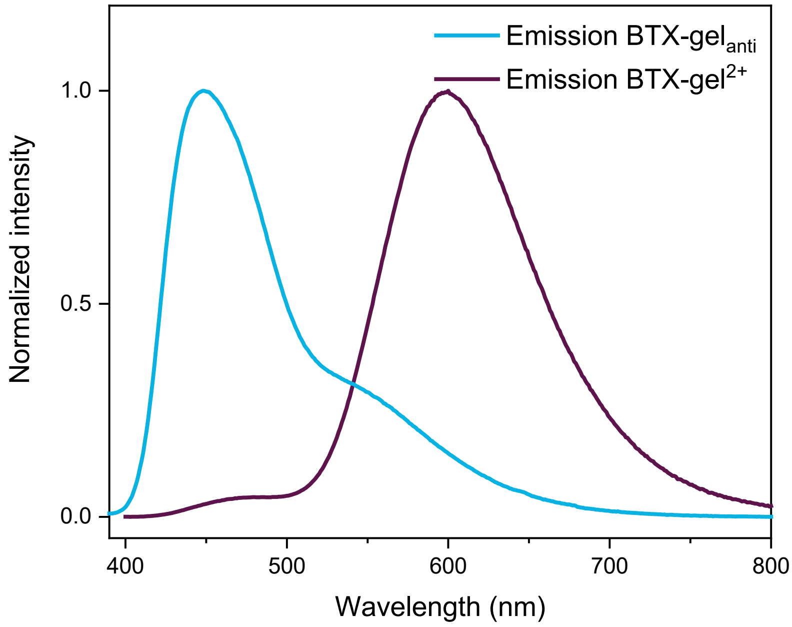

Supplementary Figure 52: Normalized fluorescence emission spectra of **BTX-gel*anti*** (λ_{ex} = 365 nm) and **BTX-gel^{2+}** (obtained by oxidation with 15 mM CAN) (λ_{ex} = 350 nm)

9 Profilometry

The line profile scans and 3D mapping of the surface topography were performed using a DektakXT Bruker profilometer with a stylus tip radius of 12.5 µm and 1 mg of stylus force. An average thickness of the polymer on the glass slide, when using a 100 µm spacer to prepare the polymer, was found to be 148.8 µm, which is in accordance with the size increase between the freshly polymerized gel and the gel in water.

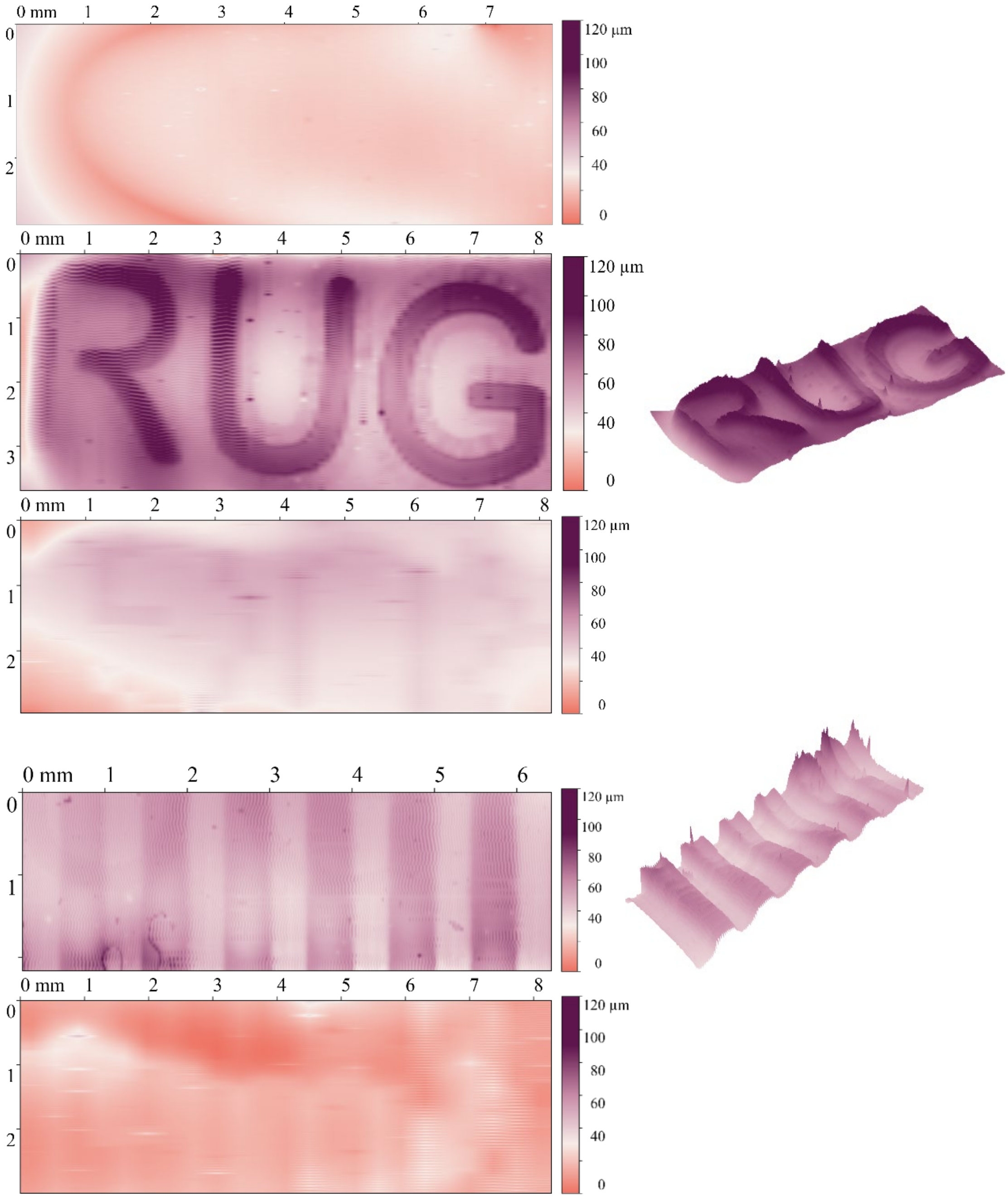

Supplementary Figure 53: 2D and 3D profiles of **BTX-gel** after applying and erasing various patterns.

10 Patterning

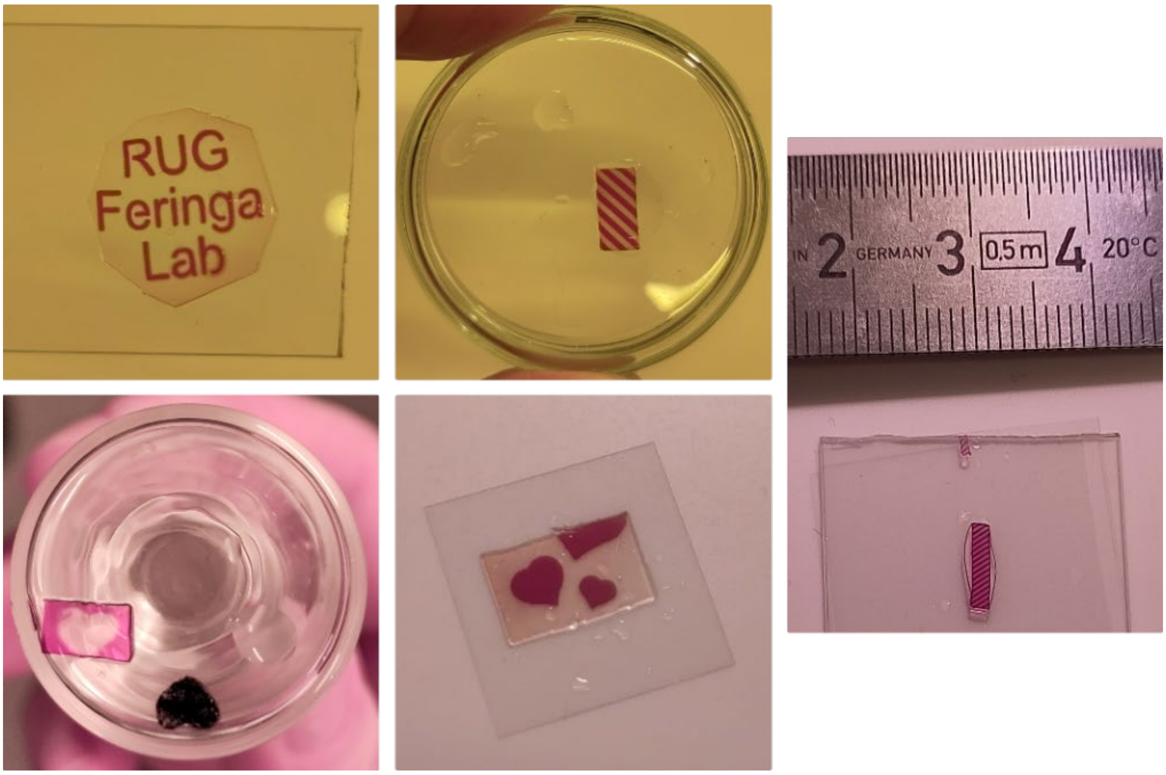

Supplementary Figure 54: Photographs of different patterns on freestanding BTX-gel (Method 1) prepared via the patterning procedure described in the Extended Data. The bottom right (heart) photomask is also visible, drawn in black marker. The other samples were made using metal photomasks. Photographs taken using a Fairphone 4 camera.

11 Supplementary References

1 Corbet, B. P., Wonink, M. B. S. & Feringa, B. L. Fast synthesis and redox switching of di- and tetra-substituted bisthioxanthylidene overcrowded alkenes. *Chem. Commun.* **57**, 7665-7668, (2021).

2 Browne, W. R., Pollard, M. M., de Lange, B., Meetsma, A. & Feringa, B. L. Reversible Three-State Switching of Luminescence: A New Twist to Electro- and Photochromic Behavior. *J. Am. Chem. Soc.* **128**, 12412-12413, (2006).

3 Hein, R., Stindt, C. N. & Feringa, B. L. Mix and Match Tuning of the Conformational and Multistate Redox Switching Properties of an Overcrowded Alkene. *J. Am. Chem. Soc.* **146**, 26275-26285, (2024).

4 Hein, R., Gisbert, Y. & Feringa, B. L. Multi-State Redox and Light-Driven Switching of Pseudorotaxanation and Cation Shuttling. *J. Am. Chem. Soc.* **147**, 13649-13657, (2025).

5 Shoup, D. & Szabo, A. Chronoamperometric current at finite disk electrodes. *J. Electroanal. Chem. Interf. Electrochem.* **140**, 237-245, (1982).

6 Paddon, C. A., Silvester, D. S., Bhatti, F. L., Donohoe, T. J. & Compton, R. G. Coulometry on the Voltammetric Timescale: Microdisk Potential-Step Chronoamperometry in Aprotic Solvents Reliably Measures the Number of Electrons Transferred in an Electrode Process Simultaneously with the Diffusion Coefficients of the Electroactive Species. *Electroanalysis* **19**, 11-22, (2007).

7 Koopmans, T. Über die Zuordnung von Wellenfunktionen und Eigenwerten zu den einzelnen Elektronen eines Atoms. *Physica* **1**, 104-113, (1934).

8 Conradie, J. A Frontier orbital energy approach to redox potentials. *J. Phys.: Conf. Ser.* **633***,* 012045.

9 Méndez-Hernández, D. D. *et al.* Simple and accurate correlation of experimental redox potentials and DFT-calculated HOMO/LUMO energies of polycyclic aromatic hydrocarbons. *J. Mol. Model.* **19**, 2845-2848, (2013).
