# Supplementary material for: Light-gated redox switching and actuation in polymer hydrogels
Source: Nat Commun. 2025 Oct 14;16:9106. doi: 10.1038/s41467-025-64123-5 (PMC12521516; doi:10.1038/s41467-025-64123-5)
Supplement: Supplementary file 3 — Description of Additional Supplementary Files [file 41467_2025_64123_MOESM3_ESM.pdf]

Supplementary Movie 1: A piece of **BTX-gel** prepared using Method 2 and cut into a square formed a 3D tube shape when saturated with water. In this movie, this tube was held with tweezers and submerged into a 30 mM solution of CAN in water. A timelapse was recorded by taking a picture every two minutes, over the course of 56 minutes. The colorless gel quickly turns purple and swells, which causes the tube to open.

Supplementary Movie 2: This video shows a piece of **BTX-gel** prepared using Method 2 that was cut into a rectangle, which in water formed a spring-like shape. When the tube was held with tweezers and submerged in a cuvette containing a 30 mM solution of CAN in water. The colorless gel quickly turns purple and swells, and the spring unfurls to form an almost straight rectangle. Then, the CAN solution is removed and first replaced with distilled water, and then with 15 mM ascorbic acid in water. The ascorbic acid reduces the dicationic BTX^{2+} back to neutral BTX, which leads to the expulsion of water and the re-coiling of the gel. To better visualize the process, the video was accelerated 160 times.

Supplementary Movie 3: For this video, a rectangular piece of **BTX-gel** that was made using Method 1 was submerged in a 30 mM solution of $FeClO_4$ in water. The gel and solution are contained in a quartz cuvette that is lain on its side on a circular holder above a 365 nm LED lamp. When the light is switched on, *syn*-folded BTX is generated on the side that is being irradiated, which is then immediately oxidized by the $Fe(ClO_4)_3$ in the solution. The BTX^{2+} that is formed leads to the swelling of the gel only on the side that is exposed to the UV light, which in turn results in the bending of the gel. The video is a timelapse recorded over the course of 40 minutes, with an image taken each two minutes.
